# Sm/Lsm Genes Provide a Glimpse into the Early Evolution of the Spliceosome

**DOI:** 10.1371/journal.pcbi.1000315

**Published:** 2009-03-13

**Authors:** Stella Veretnik, Christopher Wills, Philippe Youkharibache, Ruben E. Valas, Philip E. Bourne

**Affiliations:** 1 San Diego Supercomputer Center, University of California San Diego, La Jolla, California, United States of America; 2 Section of Ecology and Evolutionary Biology, University of California San Diego, La Jolla, California, United States of America; 3 Bioinformatics Program, University of California San Diego, La Jolla, California, United States of America; 4 Skaggs School of Pharmacy and Pharmaceutical Sciences, University of California San Diego, La Jolla, California, United States of America; University of Chicago, United States of America

## Abstract

The spliceosome, a sophisticated molecular machine involved in the removal of intervening sequences from the coding sections of eukaryotic genes, appeared and subsequently evolved rapidly during the early stages of eukaryotic evolution. The last eukaryotic common ancestor (LECA) had both complex spliceosomal machinery and some spliceosomal introns, yet little is known about the early stages of evolution of the spliceosomal apparatus. The Sm/Lsm family of proteins has been suggested as one of the earliest components of the emerging spliceosome and hence provides a first in-depth glimpse into the evolving spliceosomal apparatus. An analysis of 335 Sm and Sm-like genes from 80 species across all three kingdoms of life reveals two significant observations. First, the eukaryotic Sm/Lsm family underwent two rapid waves of duplication with subsequent divergence resulting in 14 distinct genes. Each wave resulted in a more sophisticated spliceosome, reflecting a possible jump in the complexity of the evolving eukaryotic cell. Second, an unusually high degree of conservation in intron positions is observed within individual orthologous Sm/Lsm genes and between some of the Sm/Lsm paralogs. This suggests that functional spliceosomal introns existed before the emergence of the complete Sm/Lsm family of proteins; hence, spliceosomal machinery with considerably fewer components than today's spliceosome was already functional.

## Author Summary

The spliceosome is a complex molecular machine that removes intervening sequences (introns) from mRNAs. It is unique to eukaryotes. Although prokaryotes have self-splicing introns, they completely lack spliceosomal introns and the spliceosome itself. Yet even the simplest eukaryotic organisms have introns and a rather complex spliceosomal apparatus. Little is known about how this amazing machine rapidly evolved in early eukaryotes. Here, we attempt to reconstruct a part of this evolutionary process using one of the most fundamental components of the spliceosome—the Sm and Lsm family of proteins. Using sequence and structure analysis as well as the analysis of the intron positions in Sm and Lsm genes in conjunction with a wealth of published data, we propose a plausible scenario for some aspects of spliceosomal evolution. In particular, we suggest that the Lsm family of genes could have been the first and the most essential component that allowed rudimentary splicing of early spliceosomal introns. Extensive duplications of Lsm genes and the later rise of the Sm gene family likely reflect a gradual increase in complexity of the spliceosome.

## Introduction

The modern spliceosome is a sophisticated molecular machine consisting of over 200 protein and 5 RNA components. The appearance of the spliceosome was abrupt; it is absent in prokaryotic cells, yet simple eukaryotic organisms have a rather complex spliceosome containing at least 78 proteins [1]. The question addressed here is, can we discern the steps in the evolution of the spliceosome? The Sm/Lsm family of proteins provides potential insight into this question since this family is one of the earliest pieces of the spliceosomal complex, one which stabilizes the RNA components in the “heart” of the spliceosome [2],[3]. Even though most previous studies discuss the role of Lsm (Comment #1) proteins in splicing, they perform multiple other functions in eukaryotic cells; modification and degradation, protein chaperoning and degradation, and even translation [4],[5]. Eukaryotic Sm proteins, on the other hand, are dedicated almost exclusively to splicing, but even they exhibit at least one exception [6]. Sm/Lsm counterparts exist in archaea (Sm proteins) and bacteria (Hfq protein) where no spliceosomal introns and spliceosomal apparatus has been found. Bacterial Hfq is similar to eukaryotic Lsm in its many roles in RNA/protein biogenesis [5]. Archaeal Sm-like proteins are associated with RNase P and thus likely involved in pre-tRNA processing [7]. It is possible that additional functions of Sm-like proteins in archaea are yet to be discovered. The association of Sm/Lsm proteins with all five snRNA components (U1, U2, U4, U5 and U6) of the spliceosomal complex is critical for splicing [8]. Sm/Lsm proteins assemble as multimers to form a toroid (doughnut-shaped ring) around the U-rich motif of each snRNA thus stabilizing the RNA structure and promoting the binding of other U-specific proteins to the spliceosomal RNP as they assemble [2],[4].

Structurally, each Sm/Lsm protein monomer is a small five-stranded β-barrel in which β-strands 4 and 5 are linked through a 3–10 helix to form a wide-open hinge over the rest of the barrel. These two strands are involved, on the external side, in monomer-monomer interactions to maintain the doughnut-shaped heptameric ring, and surround the RNA [2] (see PDBid 1i81; (Figure S1). The loops between β-strands 2 and 3 and between β-strands 4 and 5 (the 3–10 helix) face into the lumen of the ring, where they interact directly with the RNA. Residues within each loop and the adjacent strand form two nucleotide binding pockets (one per loop); these are among the most conserved residues in the entire structure (Fig S3). Each of the two pockets is contained within a different sequence motif. Motif I (also called SM1) includes β-sheets 1–3, while motif II (SM2) includes β-sheets 4 and 5 (1) (Pfam PFO1423, Interpro IPR001163). The loop between β-strands 3 and 4 is quite long (up to 25 residues) in some eukaryotic Sm proteins but much shorter or practically absent in Hfq, the bacterial counterpart [9] (Figure S4). The structure of the β-barrel is preserved among the three superkingdoms of life in spite of a low level of sequence identity (Figure S4). The Sm fold (b.38 in the SCOP classification) [10] is closely related to the SH3 fold (b.34), sharing the same topology, but varying structurally in the loops, and to the OB fold (b.40), with a topological permutation [11] (Figure S5). The SH3 and OB folds are small β-barrels found in many proteins. They are involved in a broad range of interactions with nucleic acids (OB), and in protein-protein interactions (SH3). These β-barrels are likely ancient and derived from a single framework from which many functions emerged, including RNA binding as described here. The Sm fold, in its shortest form, namely bacterial Hfq, exhibits approximate internal pseudo C2-symmetry; β-strands 1, 2A and 2B can be superimposed onto β-strands 3, 4 and 5 (Figure S2B). This suggests a possible even earlier (initial) duplication event in bacteria.

The ring formed by Sm/Lsm proteins around RNA can be homomeric or heteromeric. In bacteria, where there is typically only one copy of the Hfq (Sm-like) gene, the ring is homo-hexameric. In archaea, there are one or two genes and consequently one or two Sm rings each formed by homomeric components (it is also possible to have one hexameric and one heptameric homomer [12]. In eukaryotes there are a total of 8 distinct Lsm and 7 distinct Sm genes. Several types of rings exist. The most abundant and best studied are those involved in splicing. The Lsm ring involved in splicing is formed by seven distinct Lsm proteins (Lsm2–8) and the Sm ring involved in splicing is formed by 7 distinct Sm components (SmB, D1, D2, D3, E, F, G). The Sm ring interacts with the U1, U2, U4 and U5 RNA components of the spliceosome, while the Lsm ring interact in a similar manner with U6 [4],[8]. Several additional hetero heptameric rings have been identified [4] that are associated with other functions. The most prominent ring is the Lsm1–7 ring involved in mRNA decapping [13]. For the sake of clarity of presentation we will not consider the Lsm1 gene further in this paper, nor will we consider other rings besides the Lsm and Sm rings described above. Thus, while there are 8 Lsm genes, we will only consider 7 Lsm genes, Lsm2–Lsm8.

The conversion of homomeric into heteromeric complexes has occurred frequently in eukaryotic evolution, for example, the eukaryotic 20S proteasome [14], exosome [15], type II chaperonines [16] and ubiquitin-like proteins [17]. In all these examples there is extensive gene duplication and subsequent sequence divergence that results in multiple distinct paralogs all coming together to assemble into a structure quite similar or nearly identical to that constructed by their homomeric counterparts in prokaryotes. The reasons for such extravagant gene family expansions in eukaryotes are not completely understood, though heteromeric complexes represent a simple and elegant way to make more specific functional interactions among individual subunits and to establish a specific order of subunit interaction by breaking symmetry [16]. More generally it offers a means to achieve more complex regulatory mechanisms, by converting essentially a one-component system into a multi-component system [18]. We argue that in the case of Sm/Lsm rings, the recruitment into the complex spliceosomal machinery, at least in part, is responsible for creating such a large paralogous family.

What were the origins of the spliceosomal introns which triggered the development of the spliceosomal machinery? Several authors have suggested that the emergence of spliceosomal introns was caused by the introduction of self-splicing type II introns into the early eukaryotic ancestor [3],[19],[20]. The self-splicing introns then evolved into spliceosomal introns, by gradually losing their conserved RNA structural elements necessary for correct assembly and self-splicing. These elements gradually migrated from the introns into the cellular genome, becoming snRNA genes and were provided in *trans* to serve a similar role of aligning the exons during the spliceosomal process. Indeed, extensive base pairing between snRNAs and pre-mRNA in the spliceosome, which is required for the formation of tertiary RNA structure in which exons are juxtaposed [21], is similar to that found in self-splicing type II introns. In particular, the domain 5 stem-loop structure is similar to U6 RNA [22], while the ID3 step-loop structure is functionally similar to, and can be rescued by, U5 RNA [23]. Thus self-splicing introns appear to be sources of both spliceosomal introns and parts of spliceosomal machinery (RNA components). Group II self-splicing relies almost exclusively on RNA for alignment of splice junctions as well as the splicing reaction itself; the only protein component required is maturase (coded within the self-splicing intron). It has been proposed by several authors [2],[3] that Sm/Lsm proteins perform a function similar to that of maturase by reducing electrostatic repulsion between RNA components [2]. In this scenario Sm/Lsm proteins are indeed the first protein components of the developing spliceosome. This notion is further supported by the fact that an Sm/Lsm ring is formed around each snRNA; thus Sm/Lsm rings precede, functionally and temporally, most of the other spliceosomal components which are unique to each snRNA. Later, Sm/Lsm rings around snRNA begin to also serve as a structural platform that enabled additional and more specific interactions between other snRNP components [24].

Like most eukaryotic genes, Sm and Lsm genes have spliceosomal introns. The presence of the spliceosomal introns in the genes, which themselves are involved in the removal of introns, is intriguing and useful in pinpointing some evolutionary events, as we will see subsequently. It is important to bear in mind that when the intron position is conserved in orthologous genes, the parsimonious approach argues that the insertion of the intron occurred in an ancestral gene. If the identical intron position can be traced all the way back to deep branching eukaryotes, it can be argued that this intron existed in the Last Eukaryotic Common Ancestor (LECA). Identity of the intron position can be complete (when not only the position, but also the phase is conserved) or partial (when intron phases are different). The latter occurs when intron positions vary by one or two bases within DNA and is referred as intron ‘sliding,’ ‘slippage,’ or ‘frameshift.’ Clearly documented cases of intron sliding have been reported [25],[26] and even opponents of intron sliding theory admit that the phenomenon cannot be ruled out [27]. Recent work provides strong support for intron sliding and suggests it as the main mechanism for intron loss and gain [28]. The possibility of intron sliding by one base has strong statistical support [29]. The presence of introns in identical positions across multiple species may also have resulted from multiple independent insertion events into proto-splice sites [30]. Although this possibility cannot be discarded, recent studies have found that such multiple insertions are statistically infrequent events [31].

In this work we combine previously known (but disjointed) functional/structural information about Sm/Lsm proteins with new evidence coming from the phylogenetic analysis of the Sm/Lsm protein family and molecular analysis of intron positions in Sm and Lsm genes. Jointly these data point to some important events in the evolution of the early spliceosomal machinery.

Comment #1. *There are several different nomenclatures used for these proteins, hence causing confusion. The original eukaryotic proteins were coined Sm by Michael Lerner and Joan Steitz after the name of one of the patients with systemic lupus erythematosis from whose cell extracts snRNPs were immunopercipitated* [*32*]. *Lsm proteins ‘like Sm’ were called so because of their structural similarity to already identified Sm proteins* [*4*]. *The term Sm is also frequently applied to archaeal proteins with similar sequence and structure. In bacteria the protein is shorter, due to the absence of one of the internal loops; it was originally identified as virulence factor in E. coli required for phage Qβ replication, hence its name Hfq* [*33*]. *Recently an Hfq-like protein was identified in archaea M. jannaschii* [*34*], *indicating plasticity and interchangeability among Sm and Sm-like proteins. Sometimes the term Lsm is applied to the entire family* [*35*], *unfortunately this somewhat ambiguous term, which is particularly ill-suited to this paper where we discuss similarities, differences and evolutionary relationship between Sm and Lsm proteins. In this paper we refer to Sm proteins as either eukaryotic or archaeal in origin, whereas Lsm proteins are all eukaryotic. Hfq is the Lsm counterpart in bacteria. We refer jointly to eukaryotic Sm and Lsm proteins as Sm/Lsm for the sake of brevity.*

## Results

### Phylogenetic Analysis

A comparison of prokaryotic and eukaryotic genes provides evidence for a major evolutionary event early in eukaryote evolution. We collected and analyzed the sequences and gene structures of 335 Sm/Lsm genes covering 80 organisms from the three domains of life. All of the eukaryotes, with the exception of some early branching eukaryotes, have a complete set of 14 Sm/Lsm proteins (Comment #2) as compared to only one or two copies in prokaryotes.

**Figure 1:**
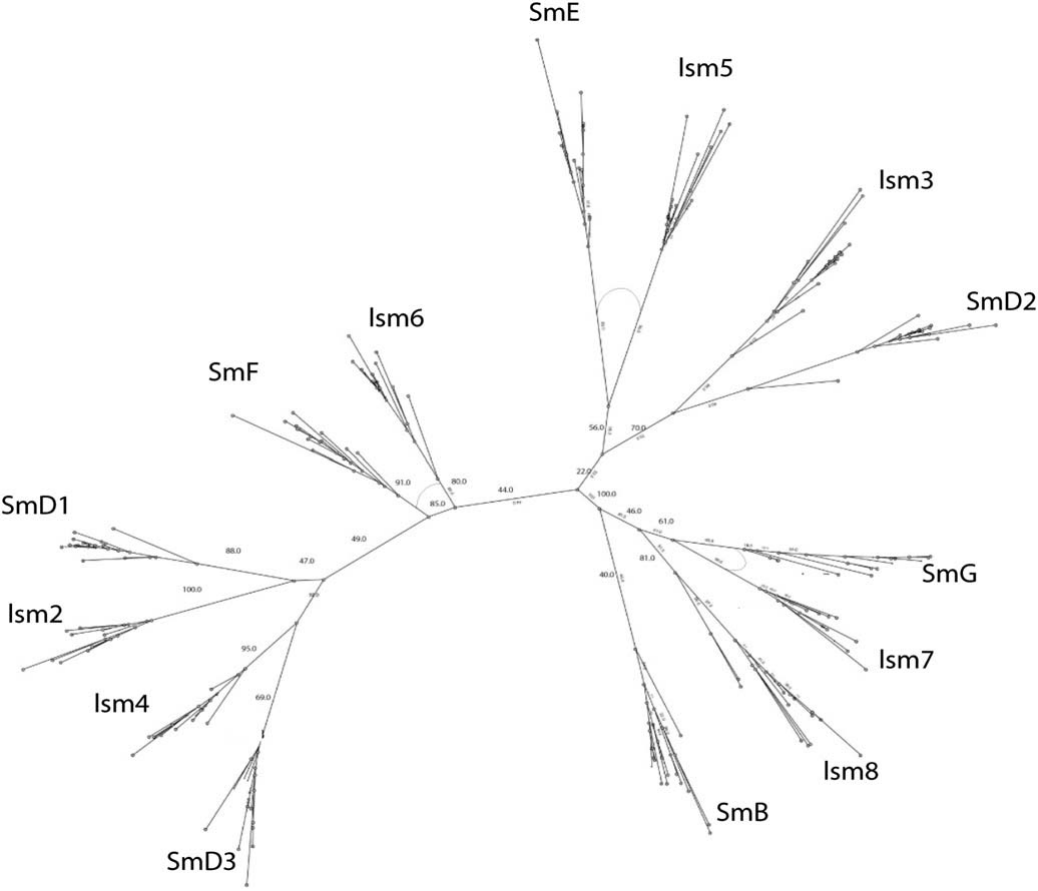
Phylogenetic tree of eukaryotic Sm/Lsm sequences. Tree was built using maximum likelihood. The values on the nodes are bootstrapping values. The arks between branches indicate that sequences in both branches share an intron in the same position.

**Figure 2 pcbi-1000315-g002:**
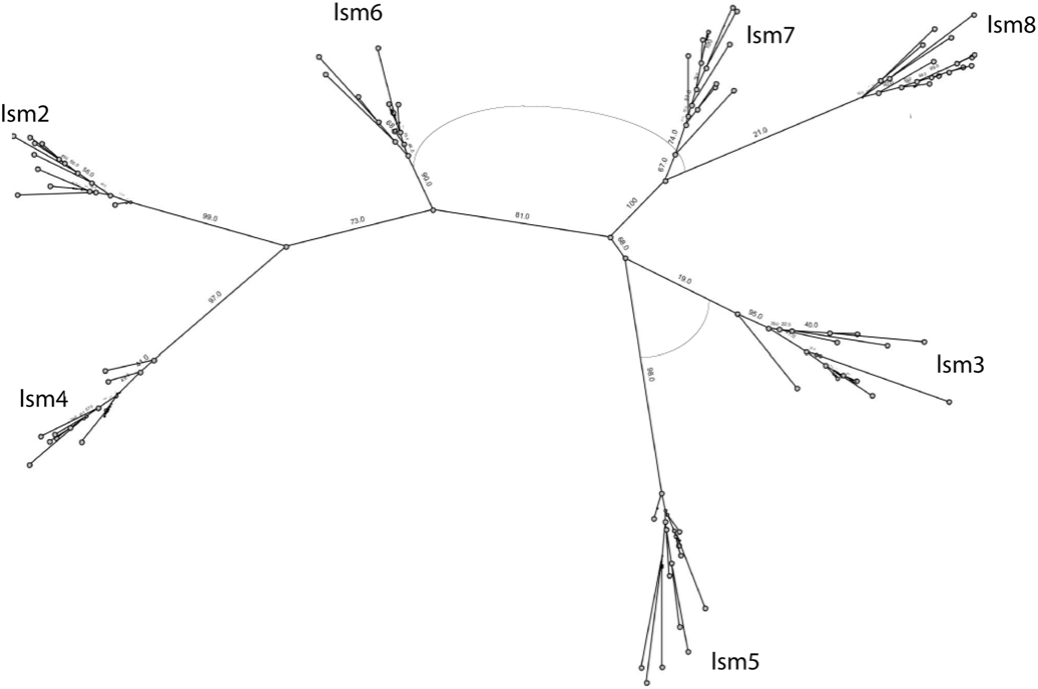
Phylogenetic tree of eukaryotic Lsm sequences. Tree was built using maximum likelihood. The values on the nodes are bootstrapping values. The arks between branches indicate that sequences in both branches share an intron in the same position.

**Figure 3:**
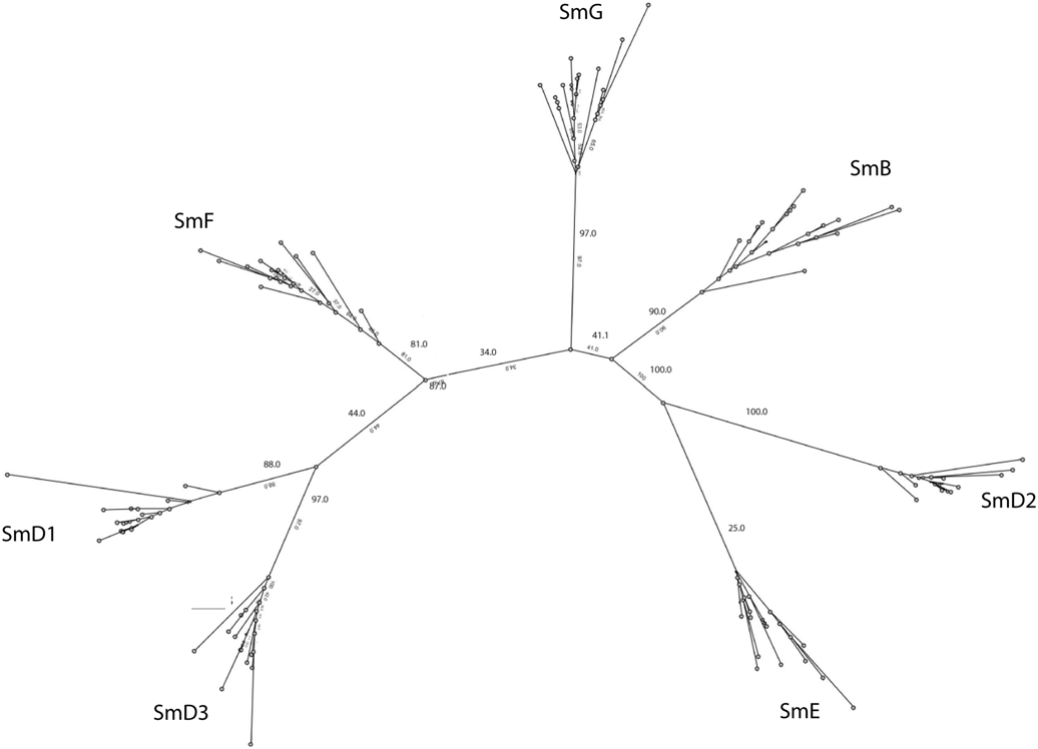
Phylogenetic tree of eukaryotic Sm sequences. Tree was built using maximum likelihood. The values on the nodes are bootstrapping values. The arks between branches indicate that sequences in both branches share an intron in the same position.

Phylogenetic analysis based on maximum likelihood (PhyMl) detects pair-wise relationships between most of the Sm-Lsm gene pairs (Figure 1). The relationships between Lsm and Sm genes are as follows: Lsm2-SmD1, Lsm3-SmD2, Lsm4-SmD3, Lsm5-SmE, Lsm6-SmF, Lsm7-SmG, Lsm8-SmB. This result suggests a scenario in which two subsequent waves of duplications occurred: the first wave resulted in 7 paralogous genes, while the second wave saw the duplication of each of the seven paralogs, bringing the total to 14 genes. To find out which of the genes, Sm or Lsm, arose on the first wave of duplication, we further constructed Lsm-only (Figure 2) and Sm-only trees (Figure 3). A tree built from Lsm genes only (Figure 2) has a similar topology to that of the eukaryotic Sm/Lsm tree (Figure 1), while the Sm-only tree indicates weaker relationship among Sm genes (Figure 3). Also some of Sm branches are longer than their Lsm counterparts: SmD3-Lsm4, Lsm5-Sm4, Lsm7-SmG, Lsm8-SmB (Figure 1). This suggests that the Sm genes have diverged further than Lsm genes, suggesting that they appear in the second wave of duplication, arising from already diversified Lsm paralogs and then proceeded to diversify further. Both waves of duplication are followed by subfunctionalization. This order of the events is further supported by functional analysis [5]. Lsm genes are involved in many RNA-processing functions, most of which evolutionary precede splicing. Sm genes, on the other hand, are almost exclusively dedicated to splicing. Although with uncertainty, it is possible to infer some order of events during the initial wave of duplication by inspecting Sm/Lsm and Lsm trees (Figures 1 and 2). One of the early duplications gave rise to the ancestor of the Lsm2–Lsm4 gene pair; the other early duplication gave rise to the ancestor of the four remaining genes: the Lsm7–Lsm8 pair and Lsm3–Lsm5 pair of genes. The pair-wise relationship among paralogs is still detectable: Lsm2–Lsm4, Lsm3–Lsm5, and Lsm7–Lsm8. The Lsm6 gene is roughly equidistant from the remaining paralogs. While many of the bootstrap values on the maximum-likelihood trees are quite high, some others are rather low, indicating an uncertainty with regards to the branching order. The same order of branching was observed in the rooted trees when eubacterial sequences were used as an outgroup (data not shown). However, use of an outgroup resulted in the reduction of the bootstrap values throughout the tree. We attribute this effect to the quality of the alignment between eubacterial and eukaryotic sequences, which is even shorter than eukaryotic alignment alone. The bacterial sequences are missing a long loop between beta-strands 3 and 4; and the second motif (SM2) - which covers beta strands 4 and 5 - is matched rather poorly. (Comment #3)

Phylogenetic trees built using Bayesian inference (Figures S6, S7, S8) are quite similar, though not identical to the trees built using a maximum likelihood approach. Sequence searches using each of the Lsm genes successfully recover their Sm counterpart with high levels of certainty (data not shown).

The type of duplication observed in Sm/Lsm genes is referred as ‘frozen duplications’(9). The number of paralogs reaches a certain number, and then stops without further expansion in any lineage. This phenomenon can be explained by the need to maintain a stochiometric balance among interacting proteins. Gene duplication resulting in gene paralogy and subsequent innovation was common during early eukaryotic evolution [18],[36]. The most extensive duplications took place in gene families involved in information processing or associated with the formation of multimeric proteins [18]. The Sm/Lsm gene family fits both these criteria; they form a multimeric ring which consists of seven distinct, but related components.

**Figure 4:**
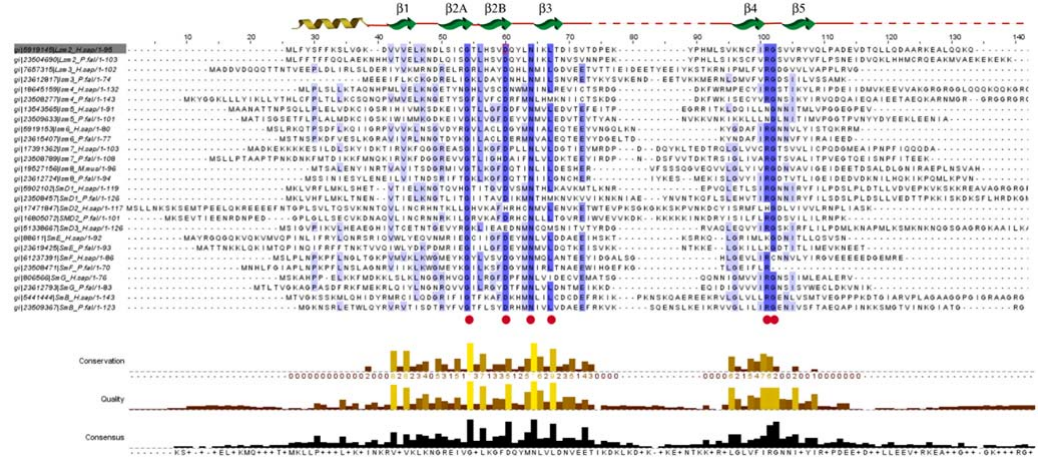
Sequence alignment of eukaryotic Sm/Lsm proteins. Overall alignment of the 14 district proteins: Lsm2–Lsm8, SmD1, SmD2, SmD3, SmE, SmF, SmG, SmB. Two representatives of each protein are shown from *H.sapiens* and *P. falciparum*. The protein secondary structure is illustrated above the alignment. The entire set of 355 sequences was aligned using ClustalW (see Text S1). Conserved positions in the alignment are shaded; positions conserved across most or all of the 355 sequences are labeled with a red dot below the alignment. Plots indicate the level of conservation, quality and consensus.

**Figure 5:**
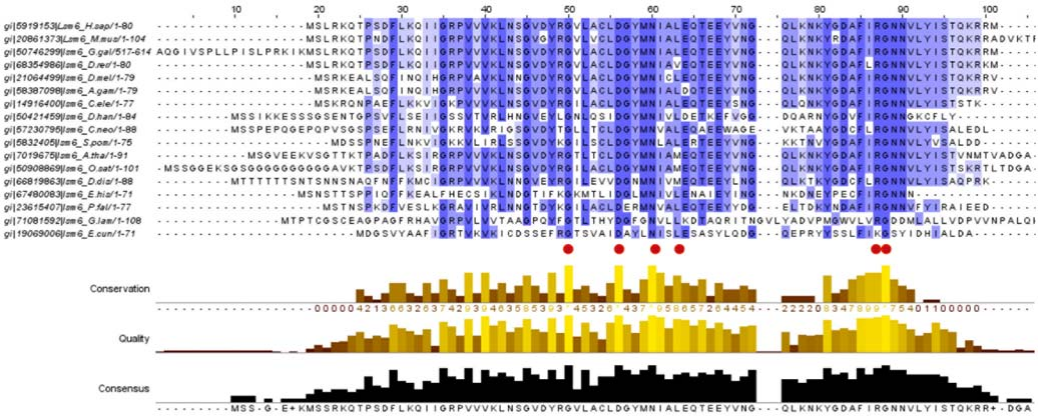
Sequence alignment of eukaryotic Lsm6 genes across the 17 eukaryotic species analyzed. (For more details see legend in Figure 4.)

Sequence analysis reveals that the Sm/Lsm gene family had nearly achieved its current configuration by the time of the last eukaryotic common ancestor (LECA) emerged. The total sequence divergence among Sm and Lsm genes before LECA (Figure 4) is 2–4 times (see ‘Calculating the level of sequence conservation’ in Text S1, Table S1) as extensive as the subsequent divergence that has taken place since the emergence of LECA approximately two and a half billion years ago (Figure 5). If we assume that the first eukaryotes appeared as early as 3 billion years ago, then the divergence that led to the LECA type of Sm/Lsm gene family took place during that half billion year period—10–20 times as rapid as the subsequent divergence in the two and a half billion years since LECA.

### Completeness of Sm and Lsm Families in Eukaryotes

We have attempted to assemble a complete set of Sm and Lsm genes for some of the eukaryotic lineages. We find that the completeness of Sm and Lsm gene families (Table 1) correlates well with the number of introns present in each genome of these eukaryotes. For example, the microsporidian *Encephalitozoon cuniculi*, , the kinetoplastid *Trypanosoma brucei* and the protist *Giardia lamblia* all have fewer introns per genome and have less regular and less complete sets of Sm/Lsm proteins than other eukaryotes (Table 1). In contrast, the amoebozoan *Dictyostelium discoideum* and the apicomplexan parasite *Plasmodium falciparum* have many introns and a nearly regular set of Sm/Lsm proteins. Incompleteness of the Sm/Lsm families in these eukaryotes is most likely due to the evolution of individual Sm/Lsm components beyond sequence recognition. Indeed most of the detected Sm and Lsm sequences, while clustering correctly, have long branches indicating extensive divergence. It is also possible that some Sm or Lsm components were lost along with the majority of the introns in these organisms during streamlining of the genome. However, most of the above parasitic organisms (as well as the nucleomorph *G. theta*) could potentially use the host's Sm/Lsm components for splicing.

**Table 1:** Catalog of Sm and Lsm genes in 6 eukaryotes from super-groups of Excavates, Chromoalveolates, and Amoebozoa.

	*T. brucei*	*G. lamblia*	*P. falciparum*	*G. theta*	*D. discoideum*	*E. histolitica*
**Lsm2**			**regular**		**regular**	**regular**
			gi|23504690		gi|66812912	gi|56471195
**SmD1**		possible	**regular**	possible	**regular**	**regular**
		gi|71071371	gi|23508457	gi|13812103	gi|66816135	gi|67479931
**Lsm3**	**regular**	possible	**regular**		**regular**	**regular**
	gi|62359545	gi|71068733	gi|23612817		gi|66818611	gi|67484430
**SmD2**	**regular**		**regular**	possible	**regular**	**regular**
	gi|62358544		gi|16805072	gi|12580778	gi|66809065	gi|67470740
**Lsm4**	possible	possible	**regular**	possible	**regular**	**regular**
	gi|70834838	gi|71071903	gi|23508277	gi|13812341	gi|66807347	gi|67481025
				gi|13812245		
**SmD3**	possible		**regular**	possible	**regular**	**regular**
	gi|62360517		gi|23613617	gi|12580710	gi|66823189	gi|67476256
**Lsm5**			**regular**	**regular**	**regular**	
			gi|23509633	gi|12580772	gi|66827081	
**SmE**	**regular**		**regular**		possible	**regular**
	gi|62360561		gi|23619425		gi|66809065	gi|67478492
**Lsm6**		possible	**regular**		**regular**	possible
		gi|71081592	gi|23615407		gi|66819863	gi|67480083
**SmF**			**regular**	**regular**	**regular**	**regular**
			gi|23508471	gi|13812032	gi|66815943	gi|67476154
**Lsm7**	possible	possible	**regular**	possible	**regular**	possible
	gi|62360185	gi|71076381	gi|23508789	gi|13812173	gi|66804897	gi|67483210
**SmG**	possible		**regular**	possible	**regular**	**regular**
	gi|70834873		gi|23612793	gi|13812173	gi|66811318	gi|67468165
						possible
						gi|67471371
**Lsm8**	possible		**regular**		**regular**	
	gi|62359836		gi|23612724		gi|90970532	
**SmB**		possible	**regular**		**regular**	**regular**
		gi|71080075	gi|23509367		gi|66823569	gi|67475017

Genes were annotated by their homology to the eukaryotes from animals, fungi and plants (see Text S1).

Genes were labeled as regular (Sm/Lsm) if they were identified as such in the NCBI database. Genes were labeled as possible (Sm/Lsm) if they were not annotated as such in the database entries, but annotation could be transferred from known genes used as a query in the PSI-BLAST search. In most cases the search yielded homologs with reliable P-value (<10^−15^). However in some cases of eukaryotes (*Dictyostelium discoideum*, *Plasmodium falciparum*, *Trypanosoma brucei*, *Entamoeba histolytica*, *Guillardia theta*, *Giardia lamblia*, and *Encephalitozoon cuniculi*) the homologs were quite remote, however after a second iteration of PSI-BLAST the P-values were in the range of 10^−4^–10^−12^. Such cases of annotation transfer should be used with caution. Some genes are absent as indicated by an empty entry.

### Conservation of Intron Positions in Eukaryotic Sm and Lsm Genes

**Figure 6:**
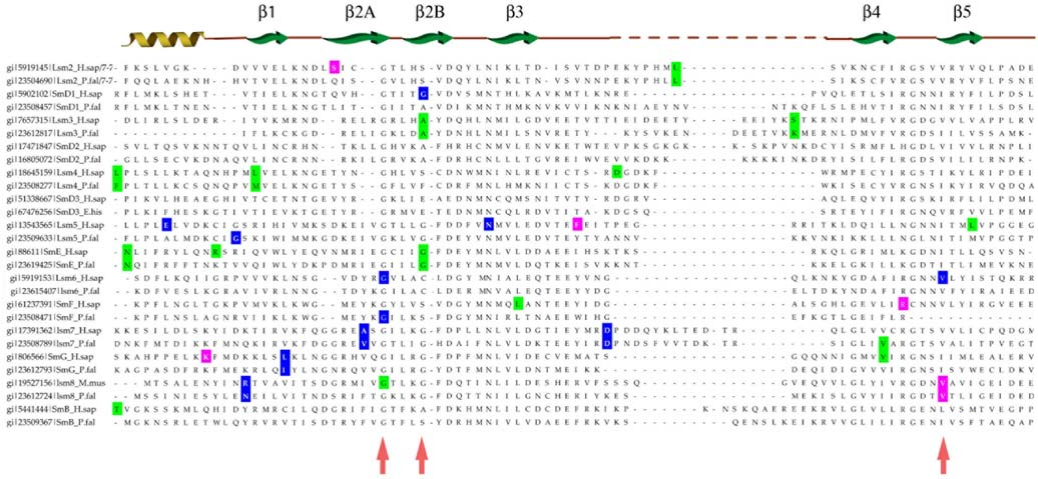
Structure of eukaryotic Sm/Lsm genes. Intron positions are marked; intron phase is indicated as follows: green - phase 0 introns; blue - phase 1 introns; magenta - phase 2 introns. Arrows indicate common introns positions shared between two or more distinct Sm/Lsm genes. Intron positions in each of 14 Sm/Lsm genes: Lsm2–Lsm8, SmD1, SmD2, SmD3, SmE, SmF, SmG, SmB. There are two representatives per family: *H.sapiens* and *P. falciparum*.

**Figure 7 pcbi-1000315-g007:**
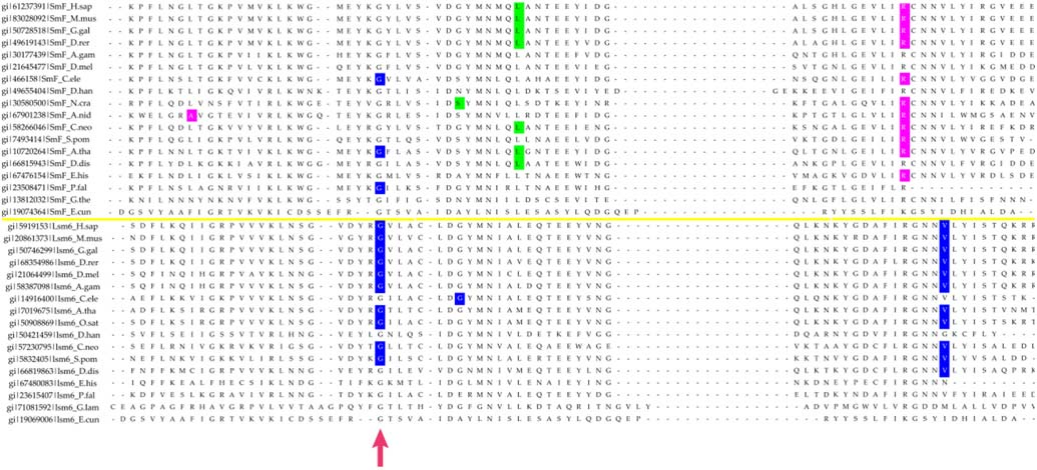
Intron positions in the Lsm6 gene and its counterpart SmF gene across eukaryotic species. Intron phase is indicated as follows: green- phase 0 introns; blue - phase 1 introns; magenta - phase 2 introns. Arrows indicate common introns positions shared between two or more distinct Sm/Lsm genes.

**Figure 8 pcbi-1000315-g008:**
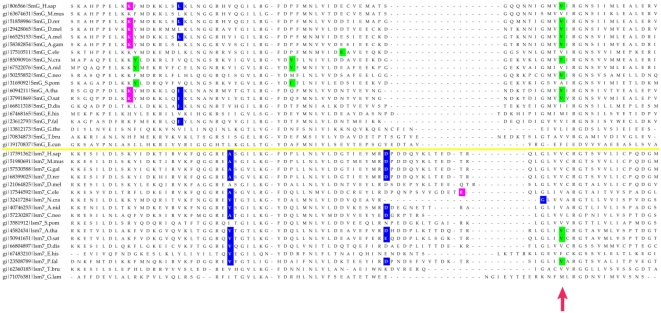
Intron positions in the Lsm7 gene and its counterpart SmG gene across eukaryotic species. Intron phase is indicated as follows: green- phase 0 introns; blue - phase 1 introns; magenta - phase 2 introns. Arrows indicate common introns positions shared between two or more distinct Sm/Lsm genes.

**Figure 9 pcbi-1000315-g009:**
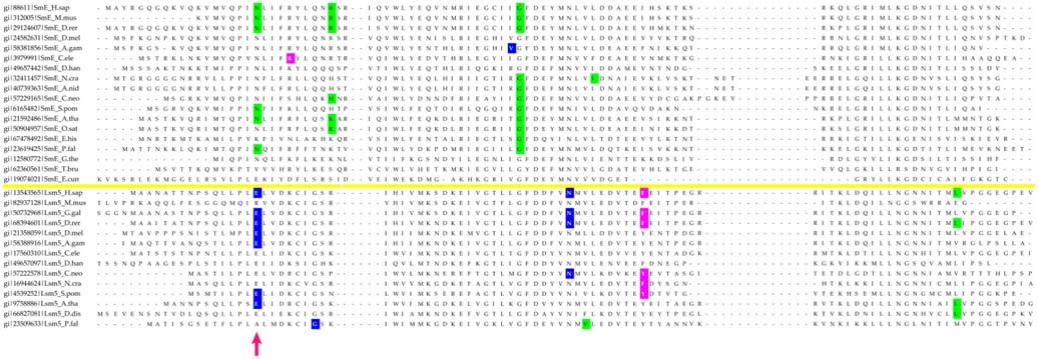
Intron positions in the Lsm5 gene and its counterpart SmE gene across eukaryotic species. Intron phase is indicated as follows: green- phase 0 introns; blue - phase 1 introns; magenta - phase 2 introns. Arrows indicate common introns positions shared between two or more distinct Sm/Lsm genes.

Most of the eukaryotic Sm/Lsm genes from the 22 species included in this study contain several introns per gene (Figures 6–13). Within each of the seven Lsm genes, and within several Sm genes, the positions of some of these introns are highly conserved across species. Sometimes this conservation extends from *D. discoideum*, *E. histolitica* and *P. falciparum*, through plants, fungi and animals (Figures 7–13). The majority of the introns which exhibit conserved position and phase across multiple species are unique to each of the 14 Sm or Lsm genes (Figure 6). Several Sm-Lsm gene pairs share identical intron positions and phases (Lsm6-SmF, Lsm7-SmG; Lsm4-SmD3, Figures 7, 8, and 12), or the same position but a different phase (Lsm5-SmE; Figure 9). Furthermore, Lsm6 and Lsm8 have two different introns in identical positions, though their phases are different (Figure 6) (Comment #4). The phase difference in all cases is 1 base.

**Figure 10 pcbi-1000315-g010:**
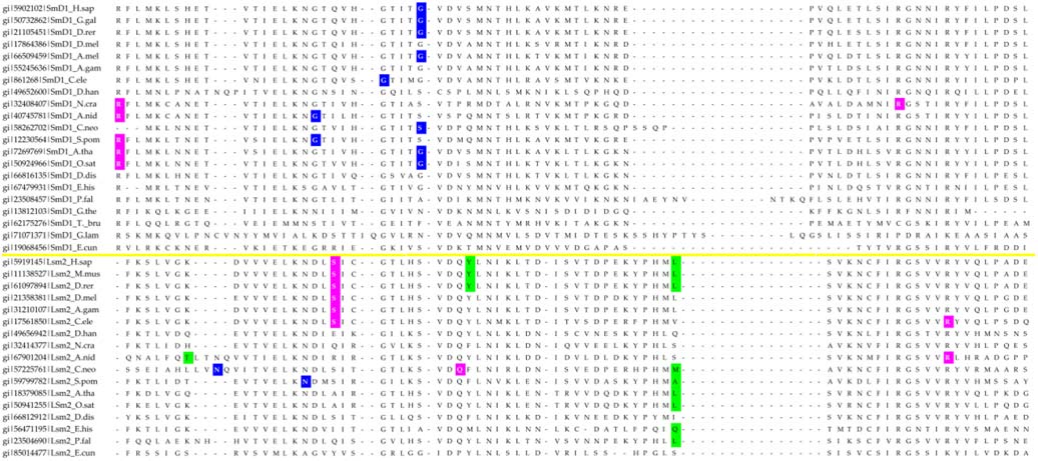
Intron positions in the Lsm2 gene and its counterpart SmD1 gene across eukaryotic species. Intron phase is indicated as follows: green- phase 0 introns; blue - phase 1 introns; magenta - phase 2 introns.

**Figure 11 pcbi-1000315-g011:**
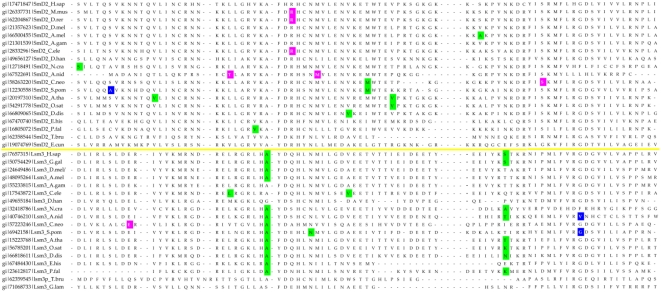
Intron positions in the Lsm3 gene and its counterpart SmD2 gene across eukaryotic species. Intron phase is indicated as follows: green - phase 0 introns; blue - phase 1 introns; magenta - phase 2 introns.

**Figure 12 pcbi-1000315-g012:**
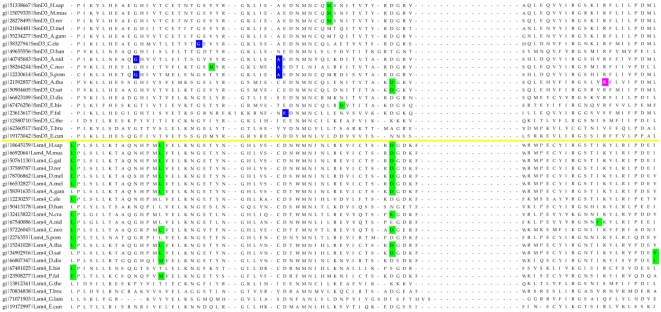
Intron positions in the Lsm4 gene and its counterpart SmD3 gene across eukaryotic species. Intron phase is indicated as follows: green - phase 0 introns; blue - phase 1 introns; magenta - phase 2 introns.

**Figure 13:**
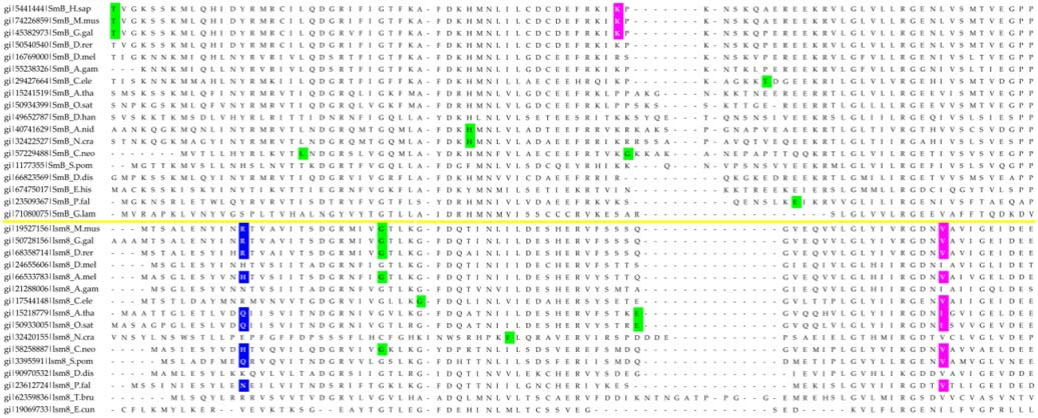
Intron positions in the Lsm8 gene and its counterpart SmB gene across eukaryotic species. Intron phase is indicated as follows: green - phase 0 introns; blue - phase 1 introns; magenta - phase 2 introns.

Comment #2. *Some of the early branching eukaryotes have a subset of 14 Sm/Lsm genes (* *Table 1*).

Comment #3. *Archael Sm genes are themselves a diverse group of sequences and consequently they cluster at several points within the eukaryotic tree rather than serving as a genuine outgroup. We believe this is because the sequence is rather short and can absorb mutations with ease, as the structure of the small beta-barrel is nearly impervious to mutations. These two features (short sequence and fully explored sequence space in both eukaryotes and archaea) make it impossible to conduct a traditional outgroup analysis using archaeal sequences.*

Comment #4. *Another intron shares position between Lsm3 and SmE, suggesting an ancestral connection between Lsm3 and Lsm5 genes. This is a more complicated scenario in which the ancestral introns are present in Lsm3 and Lsm5, transferred into the SmE genes after gene duplication and subsequently lost from Lsm5.*

## Discussion

Phylogenetic analysis indicates that the expansion of the Sm/Lsm family in eukaryotes proceeded through two distinct waves of duplication. In the first wave of duplication seven copies that later resulted in either the Lsm or the Sm genes arose through duplication from an ancestral gene. These copies then underwent extensive sequence divergence before the second wave of duplication took place. During this second wave each of the seven genes duplicated again, bringing the total number to fourteen. The extent of divergences among the paralogs is so great that many of the relationships among the seven initial paralogs cannot be reconstructed with certainty. (Figures 1 and 2). Nevertheless, some order of duplication events during the first wave can be discerned from the phylogenetic tree. The Lsm6 gene is probably the original Lsm gene, as it is roughly equidistant from the remaining six Lsm genes. Two early duplications of the ancestral Lsm gene gave rise to what was the ancestor of the two major branches – one consisting of Lsm2–Lsm4 pair, the other consisting of two pairs Lsm3–Lsm5, and Lsm7–Lsm 8 (Figure 1). Lsm7 and Lsm8 are the most closely related genes and possibly reflect the last duplication event of the first wave. A very similar scenario can be derived from trees built using Bayesian inference (Figures S6, S7, S8). The only difference is that Lsm3 and Lsm5 are more distant from each other and do not form a clear pair-wise relationship. The order of events in the second wave of duplication is impossible to predict, however the pairing between the original Lsm and the derived Sm counterpart is clearly detectable (Figure 1). The relationships between the 7 Sm genes are less clear, as would be expected if they took off and continued to evolve whereas the Lsm genes ceased to diverge.

Extensive sequence divergence observed between prokaryotic and eukaryotic orthologs is a typical feature of early stages of eukaryogenesis, where orthologous genes diverge sometimes to the point where their common ancestry is only discernable from structure [36]. The levels of divergence among the 14 paralogous Sm and Lsm genes (Figures 4 and S3) are noteworthy. Lsm genes are known to be involved in multiple cellular functions, all of which precede splicing [4],[5]. Lsm proteins, or their precursors, were very likely ‘recruited’ into the splicing machinery, further expanding the functional roles of the Lsm ring. As the seven copies of the Lsm genes diverged from each other, they were under multiple functional constraints to accommodate the different functions of the Lsm ring. The evolving splicing machinery may have required features from the Lsm ring which were in conflict with its other functions. The second wave of duplication resulted in a second ring (Sm ring) dedicated exclusively to the spliceosomal machinery (subfunctionalization). Sm genes continued to diverge extensively; some possible reasons for this divergence are discussed below. It is clear however that sufficient spliceosomal machinery was already in place to perform infrequent splicing **before** the appearance of the dedicated Sm ring.

The evidence for Lsm-only splicing comes from close examination of intron positions within the Sm and Lsm genes. It is not surprising that Sm and Lsm proteins—the components of the spliceosome—have spliceosomal introns themselves, since the vast majority of the eukaryotic genes contain introns. What is remarkable is that some of the intron positions are highly conserved across most of the 22 species studied (Figures 6–13). This level of intron conservation in these genes is greater than most other gene families. A study by Rogozin et al. [37] of intron positions in 684 orthologous genes in 8 species showed that intron positions are conserved only rarely across more than three species. In our dataset of Lsm and Sm genes many of the intron positions are conserved across 4–7 of the same species reported by Rogozin et al. (Comment #5). A recent study identified intron positions in 19 eukaryotic species [38]. A small fraction of the introns are conserved in 12–16 species; most of these introns are in genes associated with DNA/RNA processing and protein chaperoning/secretion (manuscript in preparation). Here we refer to such introns as ‘extremely conserved’, implying the conservation of intron position.

The presence of such ‘extremely conserved’ introns in the genes that are themselves key components of the spliceosome provides us with a unique opportunity to pinpoint the appearance of early functional spliceosomal introns relative to the development of the splicing machinery itself. The parsimonious approach argues that introns that exhibit highly conserved positions across multiple species are likely to stem from single intron insertion events that happened in the ancestral genes. The majority of introns which exhibit conserved position and phase across multiple species are unique to each of the 14 Sm or Lsm genes (Figure 6). This implies that these introns were introduced into the genes after the 14 separate Sm/Lsm paralogs arose by duplication in the lineage leading to the LECA. However, several Sm-Lsm gene pairs share identical intron positions and phases (Lsm6-SmF, Lsm7-SmG; Lsm4-SmD3; Figure 7, Figure 8, Figure 12) or the same position but a different phase (Lsm5-SmE; Figure 9). These introns, it can be argued, were inserted into Lsm genes before the second duplication that gave rise to Sm-Lsm pairs, indicating that some successful splicing events took place before the Sm ring was established. Further, Lsm6 and Lsm8 have two different introns in identical positions (though their phases are different) and Lsm3 and Lsm5 share one intron position (through SmE inference), arguing that some functional introns predate even the appearance of the complete set of seven Lsm genes (Figures 1 and 2). Thus, some successful splicing could occur before the formation of the Sm ring, that is, during the initial wave of duplication that led to the formation of 7 unique Lsm genes.

The fraction of introns with ‘shared’ positions between Lsm genes or between Sm genes and the corresponding Lsm counterparts is relatively small, perhaps indicating the challenges facing early spliceosomes, but more likely indicating that spliceosomal introns were likely to have been uncommon at that stage of spliceosome development. Indeed it had been recently observed that ancient paralogs share dramatically fewer intron positions than more recently formed paralogs, or even most evolutionary distant orthologs [39]. As indicated by the cases of reduced eukaryotes, which have some highly divergent or even missing Sm/Lsm genes, infrequent splicing can be accomplished with less than a full complement of these genes (Table 1) (Comment #6).

Splicing, as we suggest here, could be conducted with the Lsm ring alone, yet an additional ring (Sm) dedicated exclusively to the spliceosome arises. As we discuss above, the original Lsm ring was involved in multiple functions and its further evolution could have been hampered by multiple contradictory constraints. The appearance of a dedicated Sm ring could have allowed splicing events to become more prevalent in the cell. This alone could explain the pressure for the dedicated Sm ring. However, a more detailed look into the differences between Sm and Lsm rings gives us further insights into possible evolutionary pressures leading to the appearance of the Sm ring.

In the modern spliceosome the Lsm ring is associated with only one of the five snRNAs—namely, U6 RNA. U6 RNA is different from the four other snRNAs in several ways. First, it is the only RNA component that is transcribed by polymerase III and has a γ-monomethyl cap (U1, U2, U4 and U5 are transcribed by polymerase II and have a m3G cap). Second, U6 never leaves the nucleus: the Lsm ring is assembled in the cytoplasm and migrates to the nucleus to bind U6 RNA (which is quite different from the behavior of the other four snRNAs as we will see below). Third, U6 RNA is strikingly similar to the catalytic effector (domain 5) of the of the self-slicing group II structure [22]. Recently determined crystal structure of self-splicing group II introns further shows detailed similarity between domain 5 (DV) and U6 RNA of the spliceosome [40].

The Sm ring, on the other hand, assembles around the remaining four snRNAs - U1, U2, U4, and U5. The interactions and particularly the assembly of the Sm ring around these snRNAs is much more complex than the assembly of Lsm ring around U6 RNA. Unlike U6 RNA, U1, U2, U4 and U5 RNAs are exported into the cytoplasm, where they associate with the Sm ring and then are re-imported back into the nucleus. The assembly of the Sm ring requires assistance of a large SMN protein complex [41] and a 20S methylosome. One of the reasons for such a complicated assembly might be that the U-rich track to which the Sm ring binds is buried deep in the tertiary structure of the snRNA molecule [12] (this is different from U6, where the track is close to the 3′-end and is exposed) and some assisted refolding of the RNA by the SMN complex may be necessary.

The SMN complex associates with snRNPs through the entire cytoplasmic biogenesis on its way toward nuclear import. The interaction between Sm proteins and the SMN complex takes place through RG-rich tails on Sm proteins. Several of the Sm proteins—SmD1, SmB and SmD3 (Comment #7)—are significantly longer than their Lsm counterparts due to such RG-rich tails. These tails are also involved in the import of the Sm-RNA complex back into the nucleus [42]. It is possible that the modifications we observe in the Sm proteins, and even the appearance of the Sm ring itself, is related to the formation of the nucleus in the early eukaryotic ancestor (Comment #8). In the compartmentalized cell the spliceosomal RNA components (U1, U2, U4 U5, but not U6) consequently had to be imported back into the nucleus and the association with Sm ring was essential for their nuclear import. In total the Sm proteins underwent many changes relative to their Lsm counterparts, including changes to the electrostatic charge distribution on the surface of the ring [2],[12]—a further adjustment to the compartmentalization of the eukaryotic cell. It will be interesting to see if hitherto unrecognized features of the spliceosomal machinery can be linked to the formation of the nucleus.

Using our data and other information we can partially reconstruct a possible sequence of events in the early formation of the spliceosome with respect to Sm/Lsm proteins. As self-splicing type II introns are gradually converting into spliceosomal introns, a primitive ‘proto-spliceosome’ is at work successfully removing some introns from the transcripts (this includes removal of introns in the Sm and Lsm genes themselves). How functionally complex was the initial ‘proto-spliceosome’ is difficult to determine. Essential are the RNA components that assured formation of correct secondary structure that bring the ends of the adjacent exons into proximity. It is most likely that U6 RNA is one of the basal components of the proto-spliceosome. Were there other RNA components involved? It has recently been demonstrated that splicing can proceed with just 2 of the 5 snRNAs; namely U6 and U2 can catalyze the spliceosomal reaction [43]. In order to achieve regular splicing events, several, if not all RNA components would be needed and would be gradually added to the developing spliceosome. While currently we can not determine the order in which snRNAs were added, it is likely that all of them were associated with the Lsm ring, which stabilized electrostatic charges around the splice site.

The original Lsm ring involved in early splicing may not have yet developed its seven distinct components, but was somewhere within the first wave of duplication (since Lsm 6 and Lsm 8 as well as Lsm3 and Lsm5 have shared intron positions). Once seven unique Lsm components of the ring were formed, there was a lengthy period in which the hetero-heptameric Lsm ring does splicing alone (without the Sm ring), as evidenced by the extensive sequence divergence among the Lsm genes, as well as the accumulation of Lsm-specific introns. Sometime later a fully dedicated Sm ring appears, brought about by the duplication of each of the original Lsm components in the second wave of duplication. The appearance of the Sm ring could have been the result of the developing nucleus and the compartmentalization of the cell. Whether Sm-Lsm ‘hybrid’ rings (Comment #9) were intermediary in this process is an interesting question to ponder. Gradually the Sm proteins loose their ability to self-assemble into a ring; instead an SMN complex controls Sm ring assembly around U1, U2, U4 and U5 snRNAs; the resulting snRNPs are transported into the nucleus and become a central part of the spliceosome.

To some extent we had been lucky to have a ‘frozen event’—the association of U6 with the Lsm ring in the contemporary spliceosome—which helps us reconstruct a possible evolutionary history of Sm/Lsm proteins and strongly suggests that the Lsm ring was the original ring to be ‘recruited’ into the spliceosome and which later gave rise to the ‘dedicated’ Sm ring. In fact the use of the Sm/Lsm ring and other components from the major spliceosome in the minor spliceosome, which developed subsequent to LECA [1] and has many U12-specific components, is a good example of the continuation of the ‘recruitment’ phenomenon during the evolution of splicing. We suspect that similar scenarios of ‘recruitment’ followed by the emergence of a dedicated component through duplication exist for other spliceosomal components which were gradually added to the evolving spliceosome as its complexity increased. Whether the evolutionary history of spliceosome assembly can be teased out from the existing data remains to be seen. Notwithstanding, we hope that this type of molecular analysis, combined with structural and functional prior knowledge, can be extended to other components of the spliceosome to gain a better understanding of the events that took place at the dawn of spliceosomal introns.

Comment #5. *Since we tested a total of 22 eukaryotic species for the presence of introns in Sm/Lsm genes, some intron position exhibit an even higher level of conservation than reported by Rogozin et al.*

Comment #6. *Since most of the simple eukaryotes in* *Table 1* *are parasites, it is possible that absent Sm/Lsm genes are ‘supplemented’ by the host genome. Nevertheless, the fact that many of Sm/Lsm genes are retained in the parasite genome (albeit being highly divergent) suggests that they are used.*

Comment #7. *Interestingly, Lsm4—the proposed progenitor of SmD3—also has a RG-rich tail.*

Comment #8. *The appearance of the nucleus being a direct consequence of the appearance of spliceosomal introns was proposed recently* [*3*].

Comment #9. *The Sm-Lsm ‘hybrid’ ring is detected in association with U7snRNA which is implicated in histone pre-mRNA processing rather than splicing* [*6*].

## Methods

### Phylogenetic Analysis

The 335 sequences or the subsets of 335 sequences were aligned using ClustalW (http://align.genome.jp) under the following conditions: Scoring matrix used: BLOSUM62; Gap Open Penalty = 12 and Gap Extension Penalty = 0.1. The alignment was trimmed at N-terminus and C-terminus (to avoid regions rich in gaps); Jalview tool was used for the purpose of alignment editing (http://www.jalview.org). The final alignment used in phylogenetic analysis contains 96 residues. Two types of trees were constructed:

1. Bayesian inference trees (Figures S6, S7, S8) were constructed using the MrBayes package (http://mrbayes.csit.fsu.edu/index.php ). The data were modeled using an independent gamma distribution of the substitution rates (lset rates = gamma); amino acid substitution was modeled as mixed (aamodelpr = mixed). For the tree containing 335 sequences (not shown) the simulation was run for 7,000,000 iterations (ngen = 7,000,000) with tree sampling at every 100 generations. The average standard deviation of the split frequencies was 0.0983 at the completion of the run. The recommended average standard deviation on split frequency value should be below 0.1 and the results of our simulation are very close to this threshold (ideally it would be quite a bit lower). We believe the failure to converge further (in spite of a prolong simulation) is due to the large size of the dataset (335 sequences) combined with a short length of the alignment (96 residues). The tree with posterior probabilities indicating confidence of branching was generated after discarding the first 25% of the tree samples. For the tree containing 214 eukaryotic sequences (Figure S6) gen = 3,000,000 and ave.SD of split frequencies was 0.027; for trees containing all eukaryotic Lsm (Figure S7) and eukaryotic SM (Figure S8) sequences the simulations were run for 5,000,000 iterations and the ave SD of split frequencies were 0.019 and 0.016 correspondingly.
2. Maximum likelihood trees (Figures 1–3) were constructed by aligning the sequences using MUSCLE [44], part of the STRAP (http://www.charite.de/bioinf/strap/) suite of programs. All trees were built using PHYML [45] with the JTT model of evolution, estimated variance and gamma, and 4 substitution rate categories. PHMYL was packaged as part of Geneious [46] (http://www.geneious.com/). Each tree was bootstrapped from 100 replicates.

### Identifying Intron Positions in Sm/Lsm Genes

For all eukaryotic Sm/Lsm genes the presence of introns was checked (manually) using the NCBI Gene database. For genes containing introns, the Wise2 tool (http://www.ebi.ac.uk/Wise2/index.html ) was used to determine the exact position and the phase of each intron. TheWise2 tool was used in interactive mode under the default conditions. Both DNA and protein sequence which serve as an input into Wise2 were downloaded from NCBI. The results of Wise2 were processed manually; by marking intron positions on the protein sequences, using distinct colors to mark the intron's phase.

Additional information on sequences in this analysis as well as calculation of conservation level can be found in the Text S1.

## Supporting Information

Figure S1 RNA binding in the Sm/Lsm ring. (A) Structure of the archaeal heptameric ring (PDB code 1M8V) and its interaction with RNA. (B) Structure of the bacterial hexameric ring (PDB code 1KQ2) and its interaction with RNA. (C) Superimposition between bacterial and archaeal beta-barrels; residues colored in green are involved in interaction with RNA. (D) Superimposition between archaeal and bacterial rings: heptamer vs. hexamer.
(4.54 MB TIF)

Figure S2 Pseudo-symmetry of the Sm beta-barrel. (A) Pseudo-symmetry within the molecule: the N-terminal half is colored orange; C-terminal half is green. (B) Superimposition between two halves of the beta-barrel.
(2.17 MB TIF)

Figure S3 Alignment of 355 Sm/lsm genes from bacteria, archaea and eukaryotes. Shading of the alignment is by the level of conservation. The six most conserved residues are labeled with red dots. Secondary structure assignments are displayed above the alignment.
(7.03 MB JPG)

Figure S4 The variable loop region in Sm/lsm beta-barrel. (A) Structure of the bacterial Sm-line protein Hfq. (B) Structure of archaeal and eukaryotic Sm/lsm proteins. (C) Differences in length of the variable loop in the bacterial, archaeal and eukaryotic (SmB) small beta-barrel.
(3.38 MB TIF)

Figure S5 Relationship between SH3 and OB folds.
(3.32 MB TIF)

Figure S6 Phylogenetic tree of eukaryotic Sm and Lsm sequences reconstructed using Bayesian approach (Mr. Bayes). Tree built from eukaryotic Sm/Lsm sequences using Bayesian inference (3,000,000 iterations).
(0.78 MB TIF)

Figure S7 Phylogenetic tree of eukaryotic Sm and Lsm sequences reconstructed using Bayesian approach (Mr. Bayes). Tree built from eukaryotic Lsm sequences using Bayesian inference (2,000,000 iterations).
(0.60 MB TIF)

Figure S8 Phylogenetic tree of eukaryotic Sm and Lsm sequences reconstructed using Bayesian approach (Mr. Bayes). Tree built from eukaryotic Sm sequences using Bayesian inference B (2,000,000 iterations).
(0.56 MB TIF)

Table S1 Level of sequence conservation as calculated for inter-family alignment (divergence before LECA) and intra-family alignment (divergence since LECA).
(0.04 MB DOC)

Text S1 Supplementary Materials
(0.04 MB DOC)
