# Supplementary figures and images for: Sm/Lsm Genes Provide a Glimpse into the Early Evolution of the Spliceosome
Source: PLoS Comput Biol. 2009 Mar 13;5(3):e1000315. doi: 10.1371/journal.pcbi.1000315 (PMC2650416; doi:10.1371/journal.pcbi.1000315)

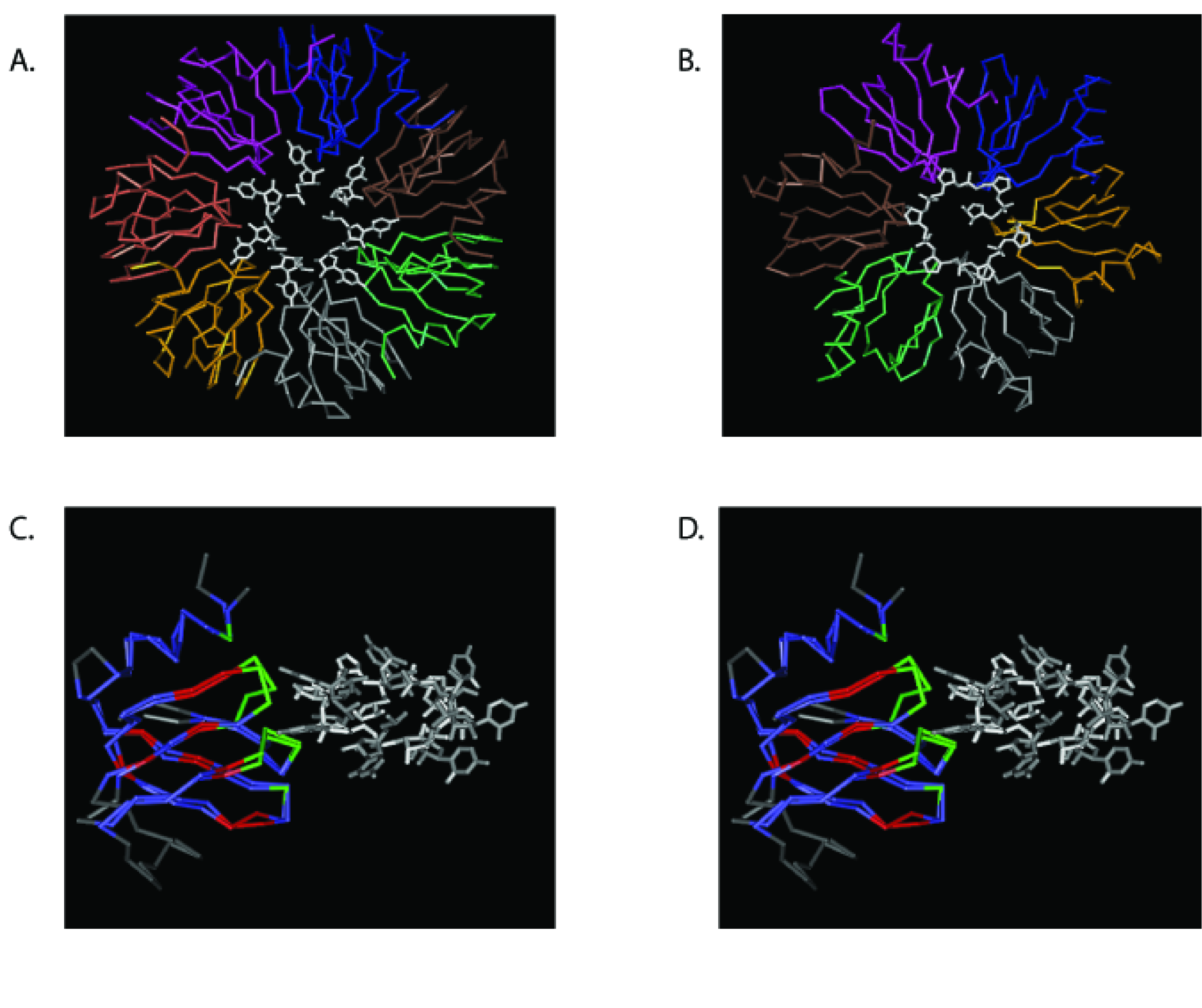

Supplement: Figure S1 — RNA binding in the Sm/Lsm ring. (A) Structure of the archaeal heptameric ring (PDB code 1M8V) and its interaction with RNA. (B) Structure of the bacterial hexameric ring (PDB code 1KQ2) and its interaction with RNA. (C) Superimposition between bacterial and archaeal beta-barrels; residues colored in green are involved in interaction with RNA. (D) Superimposition between archaeal and bacterial rings: heptamer vs. hexamer. (4.54 MB TIF) [file pcbi.1000315.s001.tif]

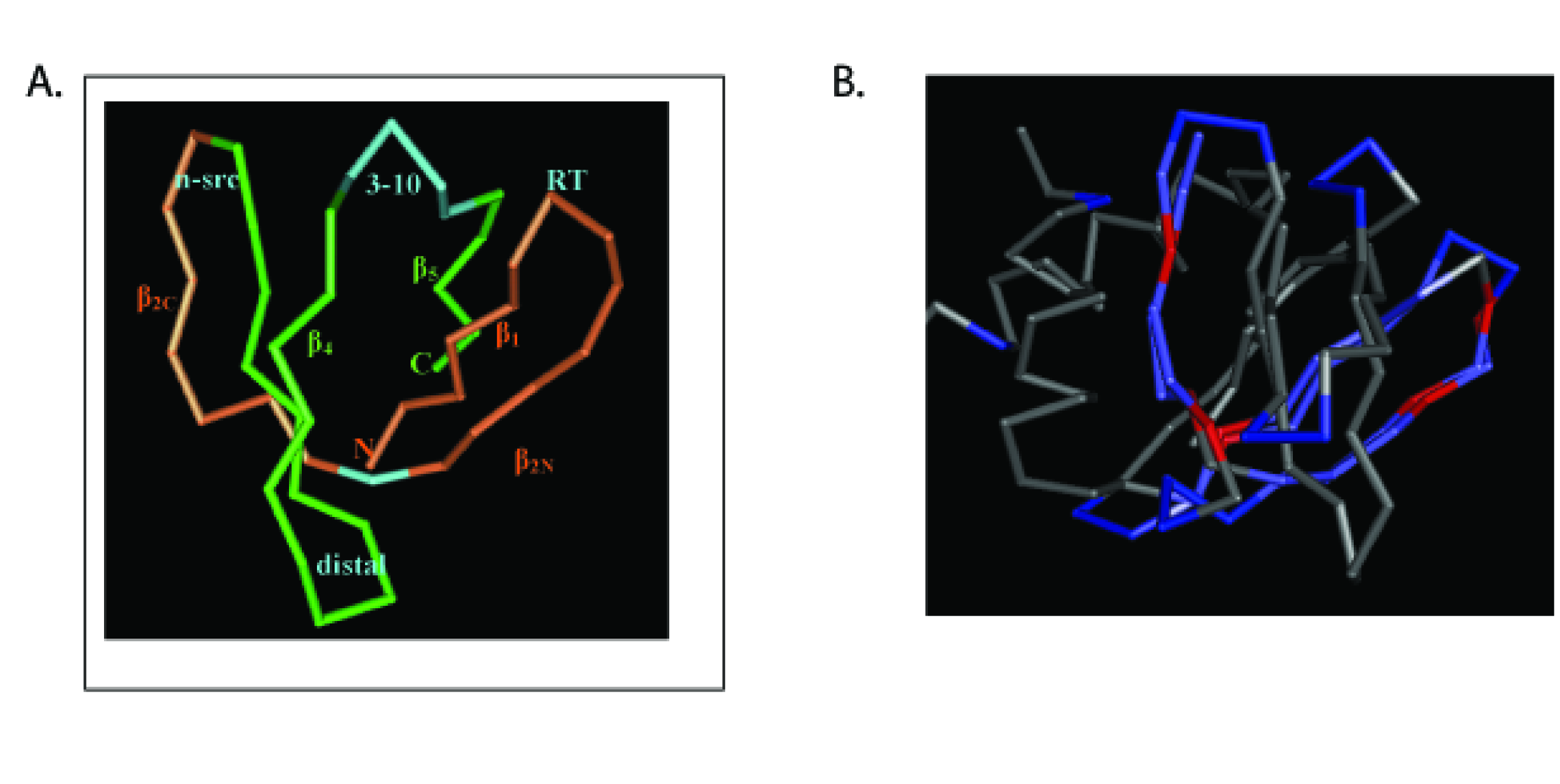

Supplement: Figure S2 — Pseudo-symmetry of the Sm beta-barrel. (A) Pseudo-symmetry within the molecule: the N-terminal half is colored orange; C-terminal half is green. (B) Superimposition between two halves of the beta-barrel. (2.17 MB TIF) [file pcbi.1000315.s002.tif]

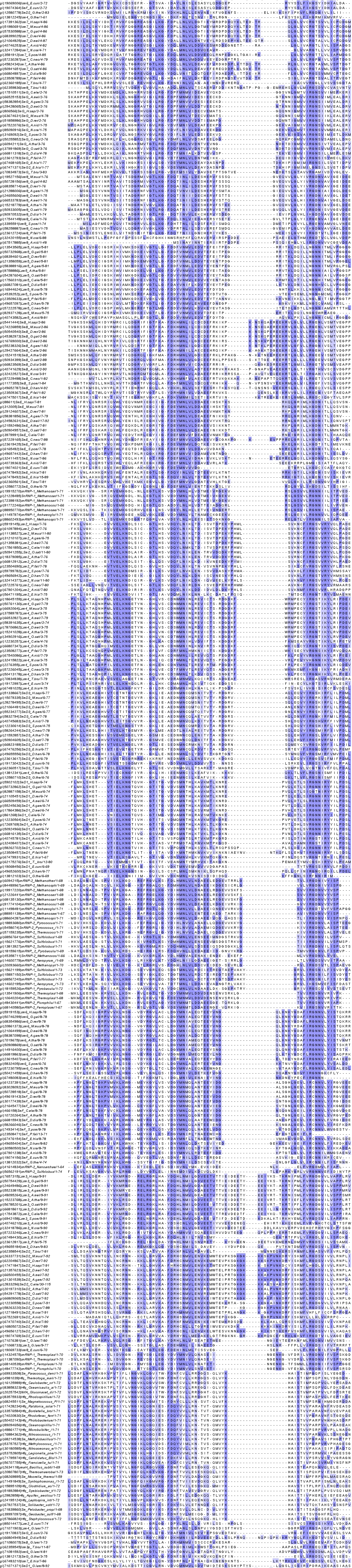

Supplement: Figure S3 — Alignment of 355 Sm/lsm genes from bacteria, archaea and eukaryotes. Shading of the alignment is by the level of conservation. The six most conserved residues are labeled with red dots. Secondary structure assignments are displayed above the alignment. (7.03 MB JPG) [file pcbi.1000315.s003.jpg]

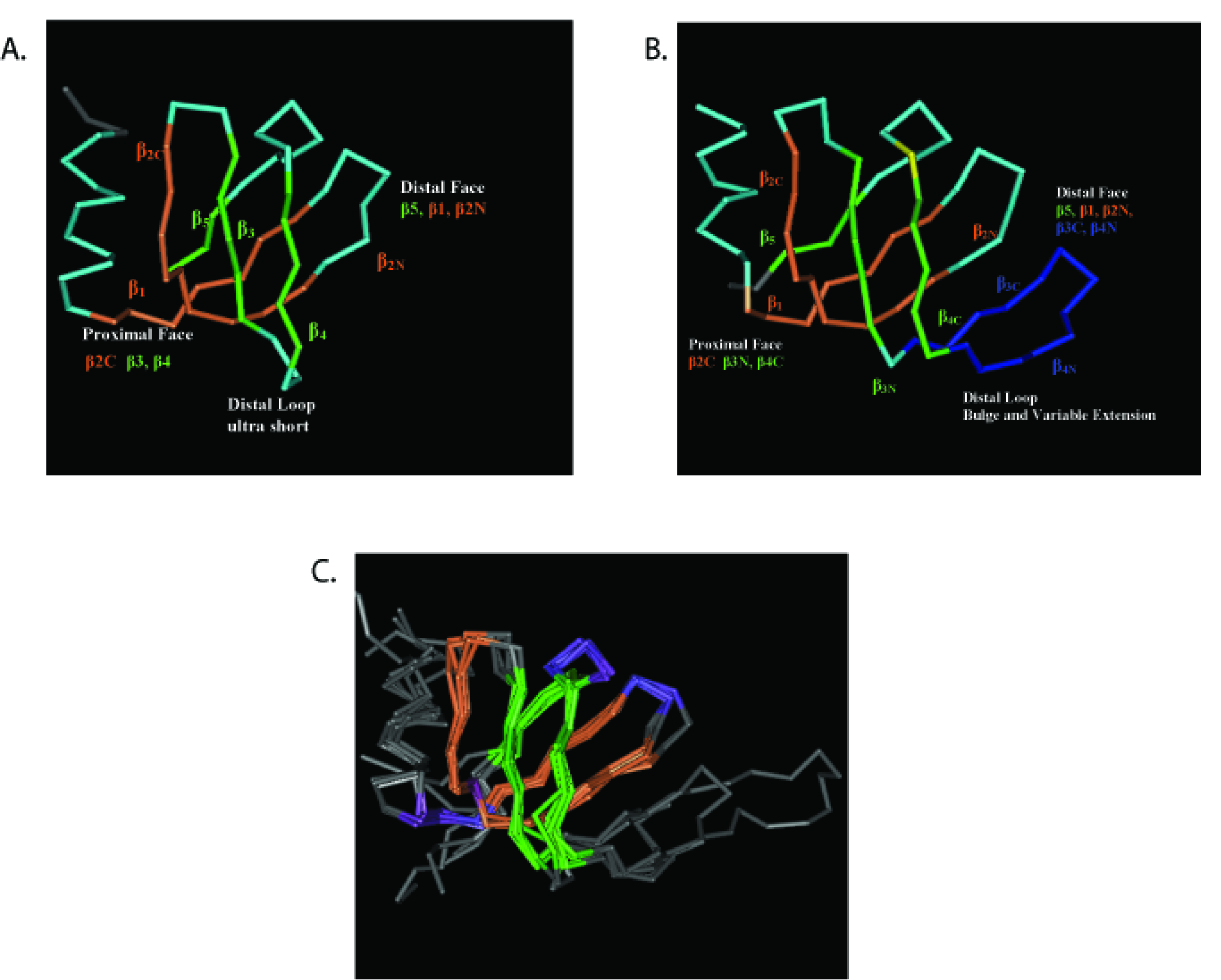

Supplement: Figure S4 — The variable loop region in Sm/lsm beta-barrel. (A) Structure of the bacterial Sm-line protein Hfq. (B) Structure of archaeal and eukaryotic Sm/lsm proteins. (C) Differences in length of the variable loop in the bacterial, archaeal and eukaryotic (SmB) small beta-barrel. (3.38 MB TIF) [file pcbi.1000315.s004.tif]

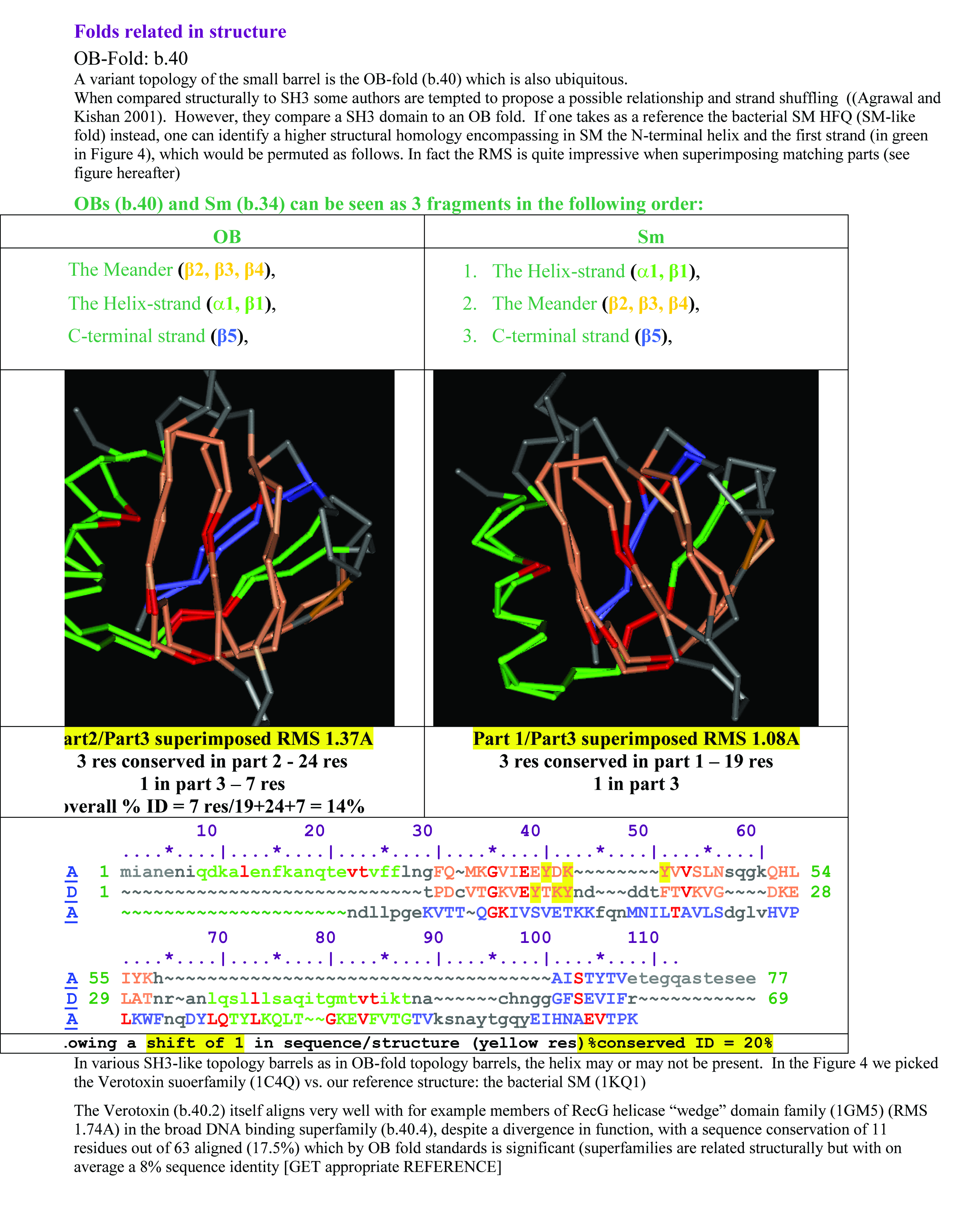

Supplement: Figure S5 — Relationship between SH3 and OB folds. (3.32 MB TIF) [file pcbi.1000315.s005.tif]

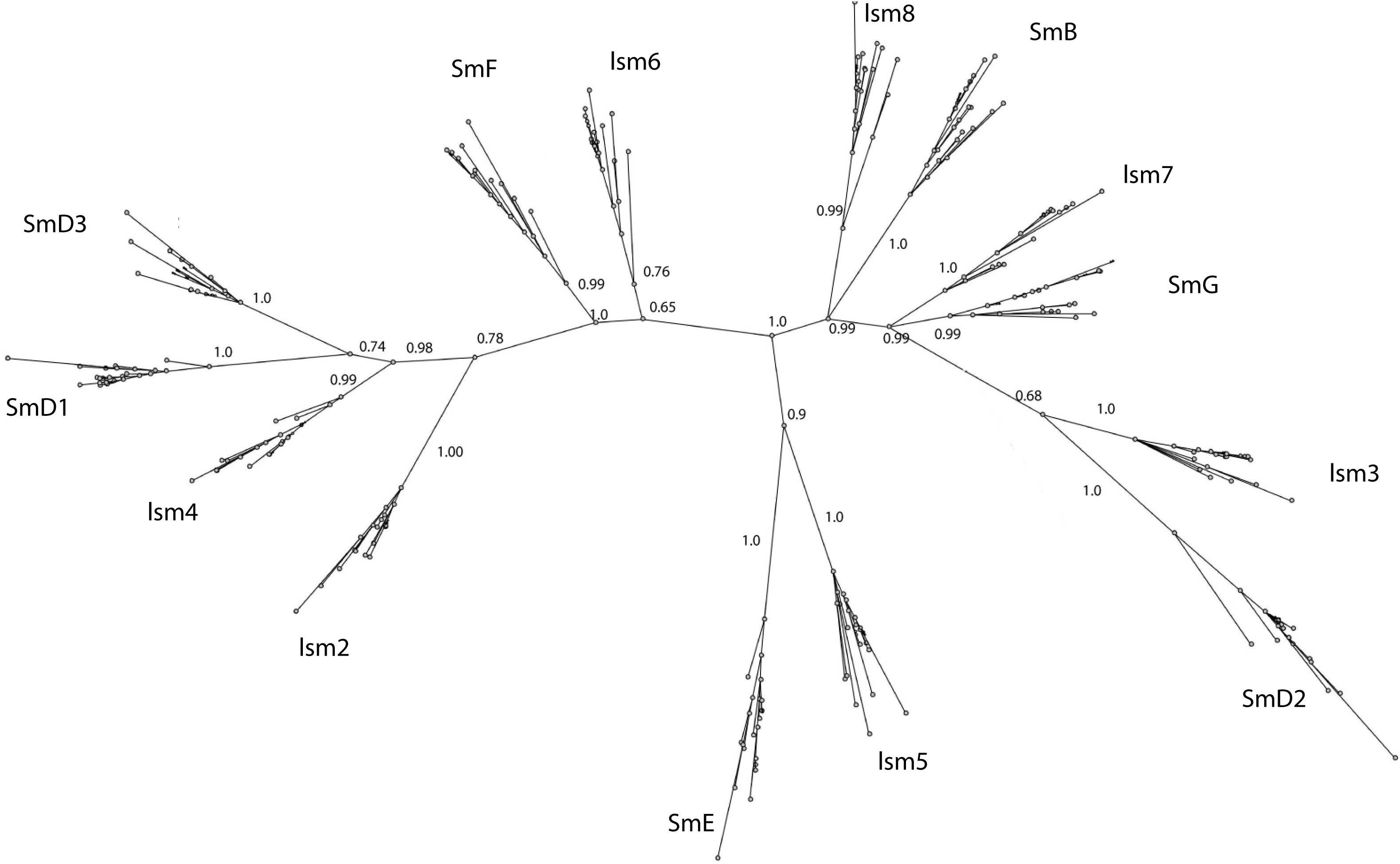

Supplement: Figure S6 — Phylogenetic tree of eukaryotic Sm and Lsm sequences reconstructed using Bayesian approach (Mr. Bayes). Tree built from eukaryotic Sm/Lsm sequences using Bayesian inference (3,000,000 iterations). (0.78 MB TIF) [file pcbi.1000315.s006.tif]

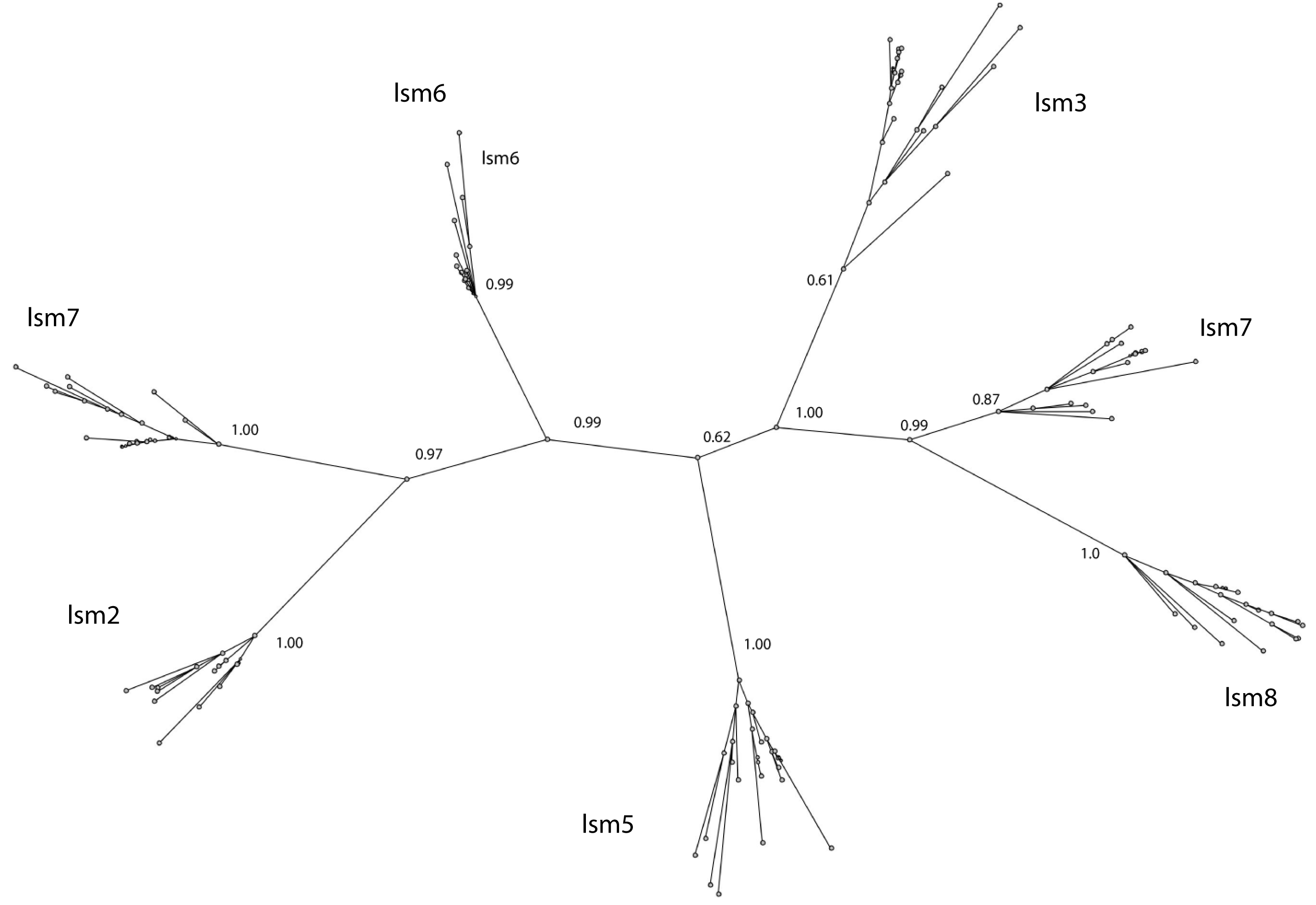

Supplement: Figure S7 — Phylogenetic tree of eukaryotic Sm and Lsm sequences reconstructed using Bayesian approach (Mr. Bayes). Tree built from eukaryotic Lsm sequences using Bayesian inference (2,000,000 iterations). (0.60 MB TIF) [file pcbi.1000315.s007.tif]

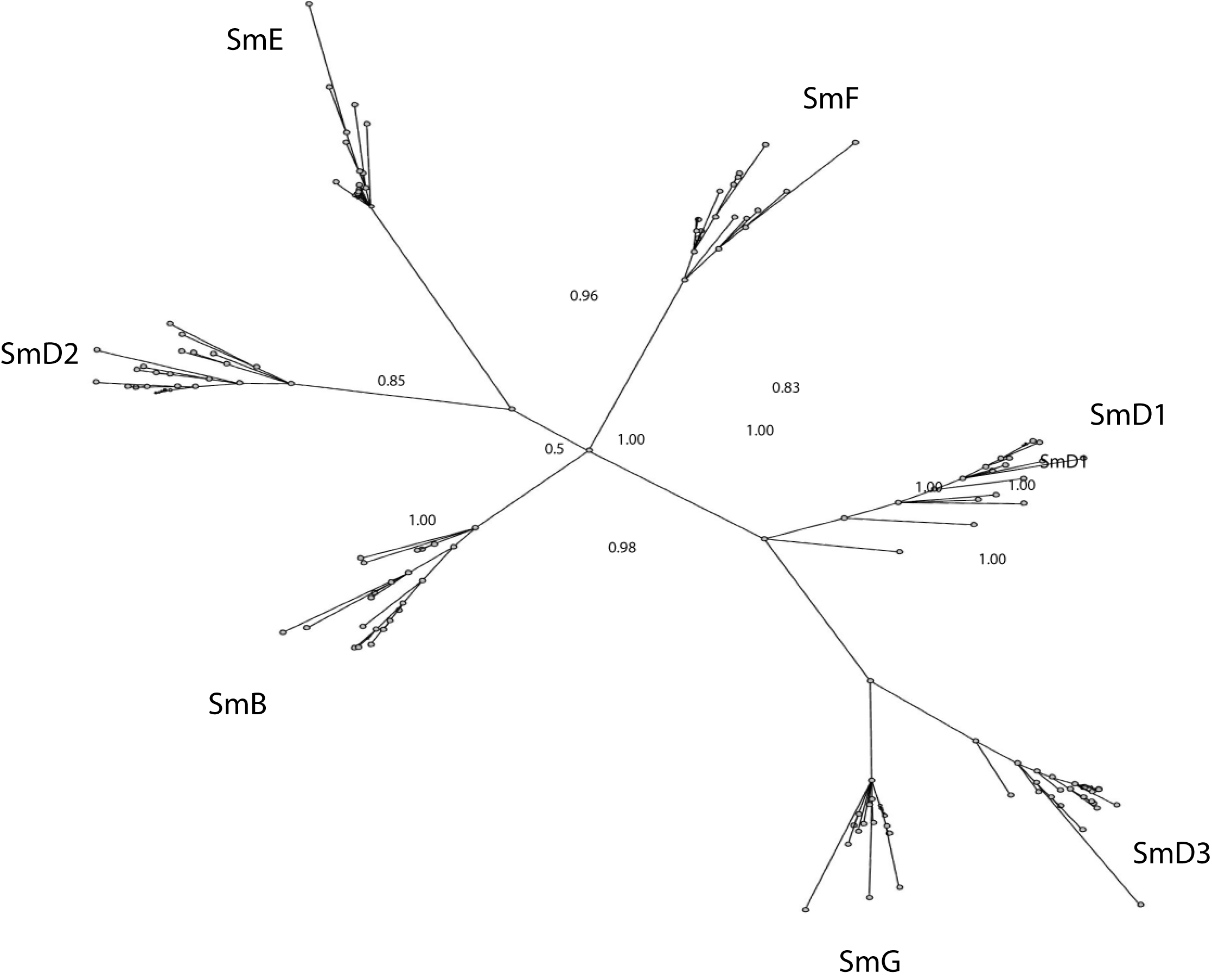

Supplement: Figure S8 — Phylogenetic tree of eukaryotic Sm and Lsm sequences reconstructed using Bayesian approach (Mr. Bayes). Tree built from eukaryotic Sm sequences using Bayesian inference B (2,000,000 iterations). (0.56 MB TIF) [file pcbi.1000315.s008.tif]
