# Supplementary material for: Sm/Lsm Genes Provide a Glimpse into the Early Evolution of the Spliceosome
Source: PLoS Comput Biol. 2009 Mar 13;5(3):e1000315. doi: 10.1371/journal.pcbi.1000315 (PMC2650416; doi:10.1371/journal.pcbi.1000315)
Supplement: Table S1 — Level of sequence conservation as calculated for inter-family alignment (divergence before LECA) and intra-family alignment (divergence since LECA). (0.04 MB DOC) [file pcbi.1000315.s009.doc]

| conservation threshold (0-1) | intra-family alignment | | | inter-family alignment | | |  |
| --- | --- | --- | --- | --- | --- | --- | --- |
|  | N conserved positions | % conserved positions | Average conservation/position | N conserved positions | % conserved positions | Average conservation/ position | ratio  intra-/inter -family |
| 0 | 95 | 100% | 0.74 | 95 | 100% | 0.43 | 1.72 |
| 0.4 | 91 | 96% | 0.75 | 41 | 43% | 0.64 | 2.62 |
| 0.5 | 83 | 88% | 0.78 | 30 | 32% | 0.71 | 3.02 |
| 0.6 | 72 | 77% | 0.82 | 25 | 27% | 0.74 | 3.16 |
| 0.7 | 53 | 56% | 0.87 | 18 | 19% | 0.78 | 3.29 |
| 0.8 | 45 | 48% | 0.90 | 12 | 13% | 0.82 | 4.05 |
| 0.9 | 25 | 27% | 0.94 | 7 | 7.4% | 0.83 | 4.13 |
