# Supplementary material for: Sm/Lsm Genes Provide a Glimpse into the Early Evolution of the Spliceosome
Source: PLoS Comput Biol. 2009 Mar 13;5(3):e1000315. doi: 10.1371/journal.pcbi.1000315 (PMC2650416; doi:10.1371/journal.pcbi.1000315)
Supplement: Text S1 — Supplementary Materials (0.04 MB DOC) [file pcbi.1000315.s010.doc]

**Supplementary Methods.**

**Collection of Sm/Lsm sequences.**

For eukaryotic Sm/Lsm genes an initial subset of sequences from *Homo sapiens* and *Mus musculus* were identified using PSI-BLAST (against nr database) with proteins known to contain the Sm fold as a query (PDB ids 1B34, 1D3B). An initial subset contained seven distinct Lsm genes: Lsm2, Lsm3, Lsm4, Lsm5, Lsm6, Lsm7 and Lsm8 and seven district Sm genes: SmB, SmD1, SmD2, SmD3, SmE, SmF and SmG.

For the remaining 20 eukaryotic species, the corresponding genes were identified using PSI-BLAST searches with human and mouse proteins as a query. The eukaryotic species used in this analysis were: *Homo sapiens, Mus musculus, Gallus gallus, Danio rerio, Drosophila melanogaster, Apis mellifera, Anopheles gambiae, Caenorhabditis elegans, Debaryomyces hansenii, Aspergillus nidulans,* *Neurospora crassa,* *Schizosaccharomyces pombe, Cryptococcus neoformans, Arabidopsis thaliana, Oryza sativa, Dictyostelium discoideum, Plasmodium falciparum, Trypanosoma brucei, Entamoeba histolytica, Guillardia theta, Giardia lamblia*, and *Encephalitozoon cuniculi*.

It should be noted that in many species the Sm and Lsm are not annotated as such in the database entries. In such cases we transferred annotation from known genes used as a query in the PSI-BLAST search. In most of the cases the search yielded homologs with reliable P-value (< 10^-15^). However in some cases of basal eukaryotes (*Dictyostelium discoideum, Plasmodium falciparum, Trypanosoma brucei, Entamoeba histolytica, Guillardia theta, Giardia lamblia*, and *Encephalitozoon cuniculi*) the homologs were quite remote, however after a second iteration of PSI-BLAST the P-values were in the range of 10^-4^– 10^-12^. Such cases of annotation transfer should be used with caution. In several basal eukaryotes we failed to identify any potential homologs and considered a gene to be absent (supplementary Figure 1). A total of 262 proteins from 22 eukaryotic species were used in this analysis.

A similar procedure was repeated for archaeal Sm/Lsm-like genes. Several archaeal genes with known structures (PDB ids 1JBM, 1LOJ, 1LNX, 1JRI, 1LJO) were used to identify archaeal genes. Twenty six species were used in this analysis: *Aeropyrum pernix, Archaeoglobus fulgidus, , Sulfolobus solfataricus, Sulfolobus tokodaii, Sulfolobus acidocaldarius, Sulfolobus neozealandicus, Pyrococcus furiosus, Pyrococcus abyssi, Pyrobaculum aerophilum, Picrophilus torridus, Ferroplasma acidarmanus, Thermoplasma volcanium, Thermoplasma acidophilum, Thermococcus kodakarensis, Methanococcus maripaludis, Methanopyrus kandleri, Methanosarcina acetivorans, Methanosarcina barkeri, Methanosarcina mazei, Methanosaeta thermophila, Methanothermobacter thermautotrophicus, Methanospirillum hungatei, Methanosphaera stadtmanae, Methanococcoides burtonii* and *Nanoarchaeum equitans*.

In many archaeal species there are two distinct Sm-like proteins: Sm1 and Sm2. We used a nomenclature of snRNP-1 and snRNP-2 to match the existing annotations. Both snRNP-1 and snRNP-2 were used if they existed. A total of 41 archaeal proteins were used in the analysis.

For bacterial proteins we used bacterial Hfq sequences with known structures (PDB ids 1KQ1, 1HK9) to identify the set of homologs from 32 bacterial species. The species used in this analysis are: **Alphaproteobacteria**: *Paracoccus denitrifican, Caulobacter crescentus, Oceanicaulis alexandrii, Gluconacetobacter diazotrophicus* and *Rhodospirillum rubrum*; **Betaproteobacteria**: *Ralstonia solanacearum, Bordetella bronchiseptica, Rhodoferax ferrireducens, Nitrosomonas europae* and*, Methylobacillus flagellatus;* **Gammaproteobacteria**: *Methylococcus capsulatus, Photobacterium profundum, Oceanospirillum sp., Microbulbifer degradans, Candidatus blochmannia, Nitrosococcus oceani, Legionella pneumophila* and *Francisella tularensis*; **Deltaproteobacteria**: *Pelobacter propionicus* and *, Geobacter sulfurreducens*; **Firmicutes**: *Syntrophomonas wolfei, Moorella thermoacetica, Staphylococcus epidermidis, Thermoanaerobacter tengcongensis, Clostridium acetobutylicum*; **Proteobacteria***:Magnetococcus sp*., **Aquificae**: *Aquifex aeolicus*, **Actinobacteria**: *Symbiobacterium thermophilum*, **Spirochaetes**: *Leptospira interrogans*, **Acidobacteria**: *Solibacter usitatus*, and **Thermotoga**:  *Thermotoga maritima*.

The entire dataset consist of 335 sequences from 80 distinct species.

The identifiers for the sequences are listed below:

Bacteria: gi_69933508,gi_68539878,gi_16125989,gi_83858323,gi_20257542,gi_74420511

gi_56551243,gi_509411,gi_14021378,gi_23347939,gi_84704741,gi_83575933

gi_84787162,gi_83751276,gi_17428234,gi_33576584,gi_34498988,gi_67543724

gi_56476105,gi_74020830,gi_30180585,gi_68214538,gi_74317551,gi_53757527

gi_16505488,gi_26251064,gi_36787838,gi_37200006,gi_21957342,gi_50402143

gi_68057212,gi_68514657,gi_52307625,gi_88858904,gi_89094656,gi_56178454

gi_71147401,gi_48861771,gi_9104867,gi_88798919,gi_71795974,gi_76884362

gi_21107932,gi_24986659,gi_78701443,gi_50085463,gi_53752805,gi_56707755

gi_71839860,gi_39997097,gi_71491849,gi_68269895,gi_2634118,gi_89209986

gi_46907521,gi_57866824,gi_20807807,gi_15895109,gi_68245511,gi_2982858

gi_51892884,gi_24195124,gi_67927537,gi_4981039

Archaea: gi_14324575,gi_11387090,gi_48477742,gi_68140539,gi_19915270,gi_21228485

gi_68211249,gi_88950770,gi_11497974,gi_13541906,gi_48430441,gi_13813927

gi_68567641,gi_18159615,gi_6094215,gi_84489967,gi_68186561,gi_68209818

gi_68135130,gi_88951139,gi_88604287,gi_18893680,gi_5458074,gi_57159235

gi_45358711,gi_14600779,gi_20093660,gi_41614834,gi_56562191,gi_15897150

gi_15621199,gi_14602195,gi_2622552

Eukaryotes: gi_17391362,gi_51980691,gi_57530588,gi_68399025,gi_21064825,gi_40746253

gi_32417284,gi_3581912,gi_57230287,gi_4582434,gi_50941651,gi_23508789

gi_7105619,gi_17510511,gi_18860007,gi_29428065,gi_66525153,gi_58382854

gi_806566,gi_63674631,gi_51858986,gi_67522076,gi_85090916,gi_6094211

gi_37991869,gi_23612793,gi_66811318,gi_67468165,gi_70834873,gi_5441444

gi_74226859,gi_50540540,gi_45382973,gi_16769000,gi_55238326,gi_29427664

gi_15241519,gi_50934399,gi_66823569,gi_40741629,gi_32422527,gi_57229488

gi_1177355,gi_49652787,gi_23509367,gi_67475017,gi_24656769,gi_66814660

gi_19170877,gi_19527156,gi_50728156,gi_68358714,gi_66533783,gi_21288006

gi_15218779,gi_50933005,gi_17544148,gi_3395591,gi_58258887,gi_23612724

gi_7657315,gi_50754429,gi_24649486,gi_55233815,gi_48095264,gi_15223768

gi_56785201,gi_66818611,gi_17543872,gi_6942158,gi_49655184,gi_57223246

gi_67484430,gi_23612817,gi_40746210,gi_32418786,gi_9837176,gi_62358544

gi_26337731,gi_62204867,gi_17471847,gi_21357623,gi_66500455,gi_21301539

gi_20197310,gi_54291778,gi_2833296,gi_66809065,gi_58263220,gi_12230558

gi_12718491,gi_67522691,gi_49656127,gi_67470740,gi_16805072,gi_12580778

gi_71076381,gi_19074769,gi_71068733,gi_19069733,gi_13543565,gi_50732968

gi_68394601,gi_21358059,gi_58388916,gi_9758886,gi_54287604,gi_17560310

gi_66827081,gi_16944624,gi_4539252,gi_23509633,gi_49657097,gi_57222578

gi_40743582,gi_82937128,gi_88611,gi_312005,gi_29124607,gi_58381856

gi_3979991,gi_21592486,gi_50904957,gi_57229165,gi_23619425,gi_6165482

gi_49657442,gi_32411457,gi_40739363,gi_67478492,gi_62360561,gi_5919145

gi_61097894,gi_11138527,gi_31210107,gi_21358381,gi_17561850,gi_50941255

gi_18379085,gi_66812912,gi_23504690,gi_59799782,gi_49656942,gi_32414377

gi_67901204,gi_57225761,gi_56471195,gi_85014477,gi_18645159,gi_50761130

gi_6692064,gi_37589787,gi_66532827,gi_58391635,gi_78706862,gi_15241028

gi_34902916,gi_12230257,gi_66807347,gi_23508277,gi_67540086,gi_32415822

gi_2276355,gi_57226045,gi_50413178,gi_70834838,gi_19172997,gi_67481025

gi_62360517,gi_13812341,gi_12580710,gi_51338667,gi_15079335,gi_28278495

gi_21064481,gi_55234277,gi_5832794,gi_40745683,gi_67523101,gi_12718393

gi_32404628,gi_12230614,gi_5689996,gi_58264244,gi_57223928,gi_21592857

gi_50904605,gi_66823189,gi_67476256,gi_49655556,gi_23613617,gi_19173042

gi_5902102,gi_82887158,gi_21105451,gi_50732862,gi_66509459,gi_55245636

gi_17864386,gi_861268,gi_7269769,gi_50924966,gi_66816135,gi_12230564

gi_40745781,gi_32408407,gi_58262702,gi_23508457,gi_67479931,gi_49652600

gi_5919153,gi_50746299,gi_68354986,gi_20861373,gi_21064499,gi_58387098

gi_7019675,gi_50908869,gi_14916400,gi_66819863,gi_23615407,gi_5832405

gi_57230795,gi_50421459,gi_67480083,gi_61237391,gi_83028092,gi_50728518

gi_49619143,gi_21645477,gi_30177439,gi_466158,gi_10720264,gi_58266046

gi_66815943,gi_7493414,gi_30580500,gi_49655404,gi_67901238,gi_67476154

gi_13812032,gi_19074156,gi_13812103,gi_71071903,gi_12580772,gi_19074021

gi_19069006,gi_62359836,gi_62360185,gi_71081592,gi_67483210,gi_66804949

**Calculating the level of sequence conservation.**

Differences in the level of sequence divergence among 14 distinct Sm/Lsm proteins (divergence before LECA) and within a single gene family (divergence since LECA) was calculated using the Scorecons server (<http://www.ebi.ac.uk/thornton-srv/databases/cgi-bin/valdar/scorecons_server.pl>). Two alignments were used for this purpose: 1. **inter-family alignment**: alignment of 14 distinct genes (Lsm2, Lsm3, Lsm4, Lsm5, Lsm6, Lsm7 and Lsm8, SmB, SmD1, SmD2, SmD3, SmE, SmF, and SmG from H.sapiens) 2. **intra-family alignment**: an alignment of Lsm6 gene across the following species (*H.sapiens*, *M.musculus, G.gallus, D.rerio, D. melanogaster, A. gambiae, C.elegans, A. thaliana, O. sativa, D. hansenii. C.neomorfans, S. pombe, D. discoideum, E.histolitica, P.falciparum, G.lamblia*, and *E.cuniculi*). Scorecons conservation level was determined under the following conditions: Scoring methods – entropic (21 types), substitution matrix - BLOSUM45, matrix transformation – linear.

An additional post-processing step was added by us to calculate the number of positions with conservation above the given threshold. Conservation threshold levels used were: 0, 0.4, 0.5, 0.6, 0.7, 0.8, 0.9 (Table S1). Number of conserved positions, percent of conserved positions and the average level of conservation per position were calculated. For each threshold level the **conservation** was defined as percent_of_conserved_positions * average_conservation_value. The ratio between intra- and inter-family **conservation** was then calculated by simply dividing the **conservation** for intra-family alignment by that of the inter-family alignment. This ratio is in the range of 1.7 to 4.1 (Table S1).
